# Current Awareness on Comparative and Functional Genomics

**DOI:** 10.1002/cfg.419

**Published:** 2005


**Comparative and Functional Genomics**
*Comp Funct Genom* 2005; **6**: 97–112
Published online in Wiley InterScience (www.interscience.wiley.com). **DOI:** 10.1002cfg.419

### Current awareness on comparative and functional genomics

CURRENT AWARENESS - USE THE LINKS ONLINE

View this bibliography online at WILEY InterScience www.interscience.wiley.com

Your gateway to Wiley journals online

The articles use crossref, the innovative multi-publisher reference linking. This enables readers to move seamlessly from the reference to the cited publication, typically located on a different server and published by a different publisher. Links to MEDLINE abstracts are also available for key journals in the biosciences.

2745

### 1 Reviews & symposia

2004. Special issue: 10th APS International Symposium "Functional Genomics of the Juxtaglomerular Apparatus". *Acta Physiol Scand* **181:** (4)
2004. Special issue: Antibodies in proteomics. *J Immunol Methods* **290:** (1-2)
2004. Special issue: Functional Genomics of Lung Disease - Second Annual Pittsburgh International Lung Conference - October 2003. *Am J Respir Cell Mol Biol* **31:** (2)
2004. Special issue: Microarrays for *Mycobacterium tuberculosis*. *Tuberculosis* **84:** (3-4)
Alba R, Fei ZJ, Payton P, Liu Y, Moore SL, Debbie P, Cohn J, D'Ascenzo M, Gordon JS, Rose JKC *et al*. 2004. ESTs, cDNA microarrays, and gene expression profiling: Tools for dissecting plant physiology and development. *Plant J* **39:** (5) 697.
Bahou WF, Gnatenko DV. 2004. Platelet transcriptome: The application of microarray analysis to platelets. *Semin Thromb Hemost* **30:** (4) 473.
Ball HJ, Hunt NH. 2004. Needle in a haystack: Microdissecting the proteome of a tissue. *Amino Acids* **27:** (1) 1.
Basu C, Halfhill MD, Mueller TC, Stewart CN. 2004. Weed genomics: New tools to understand weed biology (Review). *Trends Plant Sci* **9:** (8) 391.
Baxevanis AD. 2003. Using genomic databases for sequence-based biological discovery (Review). *Mol Med* **9:** (9-12) 185.
Benning C. 2004. Genetic mutant screening by direct metabolite analysis (Review). *Anal Biochem* **332:** (1) 1.
Brouzes E, Farge E. 2004. Interplay of mechanical deformation and patterned gene expression in developing embryos. *Curr Opin Genet Dev* **14:** (4) 367.
Brunner AM, Nilsson O. 2004. Revisiting tree maturation and floral initiation in the poplar functional genomics era (Review). *New Phytol* **164:** (1) 43.
Brunner HG, Van Driel MA. 2004. From syndrome families to functional genomics. *Nat Rev Genet* **5:** (7) 545.
Bustin SA, Dorudi S. 2004. Gene expression profiling for molecular staging and prognosis prediction in colorectal cancer. *Expert Rev Mol Diagn* **4:** (5) 599.
Celniker SE, Rubin GM. 2003. The *Drosophila melanogaster* genome. *Annu Rev Genomic Hum Genet* **4:** 89.
Church S. 2004. Advances in two-dimensional gel matching technology. *Biochem Soc Trans* **32:** (3) 511.
Conrads TP, Fusaro VA, Ross S, Johann D, Rajapakse V, Hitt BA, Steinberg SM, Kohn EC, Fishman DA, Whiteley G *et al*. 2004. High-resolution serum proteomic features for ovarian cancer detection (Review). *Endocr Relat Cancer* **11:** (2) 163.
Davis CD, Milner J. 2004. Frontiers in nutrigenomics, proteomics, metabolomics and cancer prevention (Review). *Mutat Res* **551:** (1-2) 51.
Davis CG. 2004. The role of functional genomics in selecting disease targets for antibody-based therapy (Review). *Drug Dev Res* **61:** (3) 155.
De Magalhaes JP, Toussaint O. 2004. GenAge: A genomic and proteomic network map of human ageing. *FEBS Lett* **571:** (1-3) 243.
Duncan RC, Salotra P, Goyal N, Akopyants NS, Beverley SM, Nakhasi HL. 2004. The application of gene expression microarray technology to kinetoplastid research. *Curr Mol Med* **4:** (6) 611.
Fraser AG, Marcotte EM. 2004. Development through the eyes of functional genomics. *Curr Opin Genet Dev* **14:** (4) 336.
Friedberg I, Jaroszewski L, Ye Y, Godzik A. 2004. The interplay of fold recognition and experimental structure determination in structural genomics. *Curr Opin Struct Biol* **14:** (3) 307.
Garcia A, Zitzmann N, Watson SP. 2004. Analyzing the platelet proteome. *Semin Thromb Hemost* **30:** (4) 485.
Gohil K. 2004. Functional genomics identifies novel and diverse molecular targets of nutrients *in vivo* (Review). *Biol Chem* **385:** (8) 691.
Gottlieb DM, Schultz J, Bruun SW, Jacobsen S, Sondergaard I. 2004. Multivariate approaches in plant science. *Phytochemistry* **65:** (11) 1531.
Griffin JL. 2004. Metabolic profiles to define the genome: Can we hear the phenotypes? (Review). *Philos Trans R Soc Lond B* **359:** (1446) 857.
Grimm MO, Hartmann FH, Schulz WA. 2004. Microarrays (German, English Abstract). *Urologe A* **43:** (6) 653.
Grutzmann R, Saeger HD, Luttges J, Schackert HK, Kalthoff H, Kloppel G, Pilarsky C. 2004. Microarray-based gene expression profiling in pancreatic ductal carcinoma: Status quo and perspectives (Review). *Int J Colorectal Dis* **19:** (5) 401.
Hinton JCD, Hautefort I, Eriksson S, Thompson A, Rhen M. 2004. Benefits and pitfalls of using microarrays to monitor bacterial gene expression during infection. *Curr Opin Microbiol* **7:** (3) 277.
Hirano H, Islam N, Kawasaki H. 2004. Technical aspects of functional proteomics in plants. *Phytochemistry* **65:** (11) 1487.
Honore B, Ostergaard M, Vorum H. 2004. Functional genomics studied by proteomics. *Bioessays* **26:** (8) 901.

In order to keep subscribers up-to-date with the latest developments in their field, this current awareness service is provided by John Wiley & Sons and contains newly-published material on comparative and functional genomics. Each bibliography is divided into 16 sections. 1 Reviews & symposia; 2 General; 3 Large-scale sequencing and mapping; 4 Evolutionary genomics; 5 Comparative genomics; 6 Pathways, gene families and regulons; 7 Pharmacogenomics; 8 EST, cDNA and other clone resources; 9 Functional genomics; 10 Transcriptomics; 11 Proteomics; 12 Protein structural genomics; 13 Metabolomics; 14 Genomic approaches to development; 15 Technological advances; 16 Bioinformatics. Within each section, articles are listed in alphabetical order with respect to author. If, in the preceding period, no publications are located relevant to any one of these headings, that section will be omitted.

Copyright © 2005 John Wiley & Sons, Ltd.

Joshi CP, Bhandari S, Ranjan P, Kalluri UC, Liang X, Fujino T, Samuga A. 2004. Genomics of cellulose biosynthesis in poplars (Review). *New Phytol* **164:** (1) 53.

Kell DB. 2004. Metabolomics and systems biology: Making sense of the soup. *Curr Opin Microbiol* **7:** (3) 296.

Kestler HA, Kufer R. 2004. Necessity and usefulness of bioinformatic methods for microarray data analysis (German, English Abstract). *Urologe A* **43:** (6) 669.

Khan MMK, Komatsu S. 2004. Rice proteomics: Recent developments and analysis of nuclear proteins. *Phytochemistry* **65:** (12) 1671.

Lim MS, Elenitoba-Johnson KSJ. 2004. Proteomics in pathology research (Review). *Lab Invest* **84:** (10) 1227.

Liu WM. 2004. High density DNA microarrays: Algorithms and biomedical applications. *Curr Med Chem* **11:** (16) 2143.

Lynch MD, Gill RT, Stephanopoulos G. 2004. Mapping phenotypic landscapes using DNA micro-arrays (Review). *Metab Eng* **6:** (3) 177.

Mariadason JM, Arango D, Augenlicht LH. 2004. Customizing chemotherapy for colon cancer: The potential of gene expression profiling (Review). *Drug Resist Update* **7:** (3) 209.

Meinke A, Henics T, Nagy E. 2004. Bacterial genomes pave the way to novel vaccines. *Curr Opin Microbiol* **7:** (3) 314.

Meyers BC, Scalabrin S, Morgante M. 2004. Mapping and sequencing complex genomes: Let's get physical? *Nat Rev Genet* **5:** (8) 578.

Mocellin S, Wang E, Panelli M, Pilati P, Marincola FM. 2004. DNA array-based gene profiling in tumor immunology (Review). *Clin Cancer Res* **10:** (14) 4597.

Naruse K, Hori H, Shimizu N, Kohara Y, Takeda H. 2004. Medaka genomics: A bridge between mutant phenotype and gene function (Review). *Mech Dev* **121:** (7-8) 619.

Newton RP, Brenton AG, Smith CJ, Dudley E. 2004. Plant proteome analysis by mass spectrometry: Principles, problems, pitfalls and recent developments. *Phytochemistry* **65:** (11) 1449.

Oliveri P, Davidson EH. 2004. Gene regulatory network controlling embryonic specification in the sea urchin. *Curr Opin Genet Dev* **14:** (4) 351.

Osta MA, Christophides GK, Vlachou D, Kafatos FC. 2004. Innate immunity in the malaria vector *Anopheles gambiae*: Comparative and functional genomics (Review). *J Exp Biol* **207:** (15) 2551.

Pandit SB, Balaji S, Srinivasan N. 2004. Structural and functional characterization of gene products encoded in the human genome by homology detection. *IUBMB Life* **56:** (6) 317.

Panicker RC, Huang X, Yao SQ. 2004. Recent advances in peptide-based microarray technologies. *Comb Chem High Throughput Scr* **7:** (6) 547.

Petricoin EF, Ornstein DK, Liotta LA. 2004. Clinical proteomics: Applications for prostate cancer biomarker discovery and detection. *Urol Oncol* **22:** (4) 322.

Pilate G, Dejardin A, Laurans F, Leple JC. 2004. Tension wood as a model for functional genomics of wood formation (Review). *New Phytol* **164:** (1) 63.

Poliness AE, Healey MG, Brennecke SR, Moses EK. 2004. Proteomic approaches in endometriosis research. *Proteomics* **4:** (7) 1897.

Posadas EM, Davidson B, Kohn EC. 2004. Proteomics and ovarian cancer: Implications for diagnosis and treatment: A critical review of the recent literature. *Curr Opin Oncol* **16:** (5) 478.

Reddy VS, Reddy ASN. 2004. Proteomics of calcium-signaling components in plants. *Phytochemistry* **65:** (12) 1745.

Rodland KD. 2004. Proteomics and cancer diagnosis: The potential of mass spectrometry. *Clin Biochem* **37:** (7) 579.

Rose JKC, Bashir S, Giovannnoni JJ, Jahn MM, Saravanan RS. 2004. Tackling the plant proteome: Practical approaches, hurdles and experimental tools. *Plant J* **39:** (5) 715.

Ross JS, Schenkein DP, Kashala O, Linette GP, Stec J, Symmans WF, Pusztai L, Hortobagyi GN. 2004. Pharmacogenomics (Review). *Adv Anat Pathol* **11:** (4) 211.

Sappl PG, Heazlewood JL, Millar AH. 2004. Untangling multi-gene families in plants by integrating proteomics into functional genomics. *Phytochemistry* **65:** (11) 1517.

Schmid MB. 2004. Seeing is believing: The impact of structural genomics on antimicrobial drug discovery. *Nat Rev Microbiol* **2:** (9) 739.

Shankar R, Cullinane F, Brennecke SP, Moses EK. 2004. Applications of proteomic methodologies to human pregnancy research: A growing gestation approaching delivery? *Proteomics* **4:** (7) 1909.

Stern DB, Hanson MR, Barkan A. 2004. Genetics and genomics of chloroplast biogenesis: Maize as a model system (Review). *Trends Plant Sci* **9:** (6) 293.

Suzuki M, Hayashizaki Y. 2004. Mouse-centric comparative transcriptomics of protein coding and non-coding RNAs (Review). *Bioessays* **26:** (8) 833.

Thongboonkerd V. 2004. Proteomics in nephrology: Current status and future directions. *Am J Nephrol* **24:** (3) 360.

Torday JS, Rehan VK. 2004. Deconvoluting lung evolution using functional/comparative genomics. *Am J Respir Cell Mol Biol* **31:** (1) 8.

Tsai CJ, Hubscher SL. 2004. Cryopreservation in *Populus* functional genomics (Review). *New Phytol* **164:** (1) 73.

Ussery DW, Hallin PF, Lagesen K, Wassenaar TM. 2004. tRNAs in sequenced microbial genomes (Review). *Microbiology* **150:** (6) 1603.

Wassenaar TM. 2004. Risk assessment prediction from genome sequences: Promises and dreams. *J Food Prot* **67:** (9) 2053.

Yates JR. 2004. Mass spectral analysis in proteomics. *Annu Rev Biophys Biomol Struct* **33:** 297.

Zareparsi S, Hero A, Zack DJ, Williams RW, Swaroop A. 2004. Seeing the unseen: Microarray-based gene expression profiling in vision. *Invest Ophthalmol Vis Sci* **45:** (8) 2457.

Zhang ZL, Gerstein M. 2004. Large-scale analysis of pseudogenes in the human genome. *Curr Opin Genet Dev* **14:** (4) 328.

Zhou FX, Bonin J, Predki PF. 2004. Development of functional protein microarrays for drug discovery: Progress and challenges. *Comb Chem High Throughput Scr* **7:** (6) 539.

### 3 Large-scale sequencing and mapping

Banks DJ, Porcella SF, Barbian KD, Beres SB, Philips LE, Voyich JM, DeLeo FR, Martin JM, Somerville GA, Musser JM. 2004. Progress toward characterization of the group a *Streptococcus* metagenome: Complete genome sequence of a macrolide-resistant serotype M6 strain. *J Infect Dis* **190:** (4) 727.

Bell KS, Sebaihia M, Pritchard L, Holden MTG, Hyman LJ, Holeva MC, Thomson NR, Bentley SD, Churcher LJC, Mungall K *et al*. 2004. Genome sequence of the enterobacterial phytopathogen *Erwinia carotovora* subsp *atroseptica* and characterization of virulence factors. *Proc Natl Acad Sci U S A* **101:** (30) 11105.

Bernier L. 2004. Genomics of ophiostomatoid fungi: The first two thousand genes. *Phytoprotection* **85:** (1) 39.

Bruggemann H, Henne A, Hoster F, Liesegang H, Wiezer A, Strittmatter A, Hujer S, Durre P, Gottschalk G. 2004. The complete genome sequence of *Propionibacterium acnes*, a commensal of human skin. *Science* **305:** (5684) 671.

Chien MC, Morozova I, Shi SD, Sheng HT, Chen J, Gomez SM, Asamani G, Hill K, Nuara J, Feder M *et al*. 2004. The genomic sequence of the accidental pathogen *Legionella pneumophila*. *Science* **305:** (5692) 1966.

Grozdanov L, Raasch C, Schulze E, Sonnenborn U, Gottschalk G, Hacker J, Dobrindt U. 2004. Analysis of the genome structure of the nonpathogenic probiotic *Escherichia coli* strain Nissle 1917. *J Bacteriol* **186:** (16) 5432.

Jaffe JD, Stange-Thomann N, Smith C, DeCaprio D, Fisher S, Butler J, Calvo S, Elkins T, Fitzgerald MG, Hafez N *et al*. 2004. The complete genome and proteome of *Mycoplasma mobile*. *Genome Res* **14:** (8) 1447.

Khorasani MZ, Hennig S, Imre G, Asakawa S, Palczewski S, Berger A, Hori H, Naruse K, Mitani H, Shima A *et al*. 2004. A first generation physical map of the medaka genome in BACs essential for positional cloning and clone-by-clone based genomic sequencing. *Mech Dev* **121:** (7-8) 903.

Kim IC, Kweon HS, Kim YJ, Kim CB, Gye MC, Lee WO, Lee YS, Lee JS. 2004. The complete mitochondrial genome of the javeline goby *Acanthogobius hasta* (Perciformes, Gobiidae) and phylogenetic considerations. *Gene* **336:** (2) 147.

Laurentino EC, Ruiz JC, Fazelinia G, Myler PJ, Degrave W, Alves-Ferreira M, Ribeiro JMC, Cruz AK. 2004. A survey of *Leishmania braziliensis* genome by shotgun sequencing. *Mol Biochem Parasitol* **137:** (1) 81.

Magrini V, Warren WC, Wallis J, Goldman WE, Xu J, Mardis ER, McPherson JD. 2004. Fosmid-based physical mapping of the *Histoplasma capsulatum* genome. *Genome Res* **14:** (8) 1603.

Copyright © 2005 John Wiley & Sons, Ltd. 

Song YJ, Tong ZZ, Wang J, Wang L, Guo ZB, Han YP, Zhang JG, Pei DC, Zhou DS, Qin H *et al*. 2004. Complete genome sequence of *Yersinia pestis* strain 91001, an isolate avirulent to humans. *DNA Res* **11:** (3) 179.

### 4 Evolutionary genomics

Armbrust EV, Berges JA, Bowler C, Green BR, Martinez D, Putnam NH, Zhou SG, Allen AE, Apt KE, Bechner M *et al*. 2004. The genome of the diatom *Thalassiosira pseudonana*: Ecology, evolution, and metabolism. *Science* **306:** (5693) 79.

Boussau B, Karlberg EO, Frank AC, Legault BA, Andersson SGE. 2004. Computational inference of scenarios for α-proteobacterial genome evolution. *Proc Natl Acad Sci U S A* **101:** (26) 9722.

Emes RD, Riley MC, Laukaitis CM, Goodstadt L, Karn RC, Ponting CP. 2004. Comparative evolutionary genomics of androgen-binding protein genes. *Genome Res* **14:** (8) 1516.

Frenkel FE, Chaley MB, Korotkov EV, Skryabin KG. 2004. Evolution of tRNA-like sequences and genome variability. *Gene* **335:** 57.

Genschel U. 2004. Coenzyme A biosynthesis: Reconstruction of the pathway in archaea and an evolutionary scenario based on comparative genomics. *Mol Biol Evol* **21:** (7) 1242.

Grover CE, Kim HR, Wing RA, Paterson AH, Wendel JF. 2004. Incongruent patterns of local and global genome size evolution in cotton. *Genome Res* **14:** (8) 1474.

Holden MTG, Feil EJ, Lindsay JA, Peacock SJ, Day NPJ, Enright MC, Foster TJ, Moore CE, Hurst L, Atkin R *et al*. 2004. Complete genomes of two clinical *Staphylococcus aureus* strains: Evidence for the rapid evolution of virulence and drug resistance. *Proc Natl Acad Sci U S A* **101:** (26) 9786.

Hong SH, Kim TY, Lee SY. 2004. Phylogenetic analysis based on genome-scale metabolic pathway reaction content. *Appl Microbiol Biotechnol* **65:** (2) 203.

Kouvelis VN, Ghikas DV, Typas MA. 2004. The analysis of the complete mitochondrial genome of *Lecanicillium muscarium* (synonym *Verticillium lecanii*) suggests a minimum common gene organization in mtDNAs of Sordariomycetes: Phylogenetic implications. *Fungal Genet Biol* **41:** (10) 930.

Liu H, Peng JP, Yang J, Sun LL, Chen SX, Jin Q. 2004. Analysis of components of conserved "backbone sequences" among genomes of *Shigella* spp. strains. *Chin Sci Bull* **49:** (2) 152.

Sebat J, Lakshmi B, Troge J, Alexander J, Young J, Lundin P, Maner S, Massa H, Walker M, Chi MY *et al*. 2004. Large-scale copy number polymorphism in the human genome. *Science* **305:** (5683) 525.

Telleria J, Barnabe C, Hide M, Banuls AL, Tibayrenc M. 2004. Predominant clonal evolution leads to a close parity between gene expression profiles and subspecific phylogeny in *Trypanosoma cruzi*. *Mol Biochem Parasitol* **137:** (1) 133.

Zheng JF, Liu GR, Zhu WF, Zhou YG, Liu SL. 2004. Phylogenetic clusters of rhizobia revealed by genome structures. *Sci China C Life Sci* **47:** (3) 268.

Zhong SB, Khodursky A, Dykhuizen DE, Dean AM. 2004. Evolutionary genomics of ecological specialization. *Proc Natl Acad Sci U S A* **101:** (32) 11719.

Zhou DS, Han YP, Song Y, Tong ZZ, Wang J, Guo ZB, Pei DC, Pang X, Zhai JH, Li M *et al*. 2004. DNA microarray analysis of genome dynamics in *Yersinia pestis*: Insights into bacterial genome microevolution and niche adaptation. *J Bacteriol* **186:** (15) 5138.

### 5 Comparative genomics

Bastien O, Lespinats S, Roy S, Metayer K, Fertil B, Codani JJ, Marechal E. 2004. Analysis of the compositional biases in *Plasmodium falciparum* genome and proteome using *Arabidopsis thaliana* as a reference. *Gene* **336:** (2) 163.

Boore JL, Medina M, Rosenberg LA. 2004. Complete sequences of the highly rearranged molluscan mitochondrial genomes of the scaphopod *Graptacme eborea* and the bivalve *Mytilus edulis*. *Mol Biol Evol* **21:** (8) 1492.

Chen T, Hosogi Y, Nishikawa K, Abbey K, Fleischmann RD, Walling J, Duncan MJ. 2004. Comparative whole-genome analysis of virulent and avirulent strains of *Porphyromonas gingivalis*. *J Bacteriol* **186:** (16) 5473.

Coulson RMR, Hall N, Ouzounis CA. 2004. Comparative genomics of transcriptional control in the human malaria parasite *Plasmodium falciparum*. *Genome Res* **14:** (8) 1548.

Darling ACE, Mau B, Blattner FR, Perna NT. 2004. Mauve: Multiple alignment of conserved genomic sequence with rearrangements. *Genome Res* **14:** (7) 1394.

Ferretti JJ, Ajdic D, McShan WM. 2004. Comparative genomics of streptococcal species. *Indian J Med Res* **119:** (Suppl) 1.

Francki MG, Mullan DJ. 2004. Application of comparative genomics to narrow-leafed lupin (*Lupinus angustifolius* L.) using sequence information from soybean and *Arabidopsis*. *Genome* **47:** (4) 623.

Huynen MA, Snel B, Van Noort V. 2004. Comparative genomics for reliable protein-function prediction from genomic data. *Trends Genet* **20:** (8) 340.

Kellis M, Patterson N, Birren B, Berger B, Lander ES. 2004. Methods in comparative genomics: Genome correspondence, gene identification and regulatory motif discovery. *J Comput Biol* **11:** (2-3) 319.

Khaitovich P, Muetzel B, She XW, Lachmann M, Hellmann I, Dietzsch J, Steigele S, Do HH, Weiss G, Enard W *et al*. 2004. Regional patterns of gene expression in human and chimpanzee brains. *Genome Res* **14:** (8) 1462.

Kohn LM. 2004. Applying comparative genomics to plant disease epidemiology (French). *Phytoprotection* **85:** (1) 45.

Koide T, Zaini PA, Moreira LM, Vencio RZN, Matsukuma AY, Durham AM, Teixeira DC, El-Dorry H, Monteiro PB, Da Silva ACR *et al*. 2004. DNA microarray-based genome comparison of a pathogenic and a nonpathogenic strain of *Xylella fastidiosa* delineates genes important for bacterial virulence. *J Bacteriol* **186:** (16) 5442.

Li M, Rosenshine I, Tung SL, Wang XH, Friedberg D, Hew CL, Leung KY. 2004. Comparative proteomic analysis of extracellular proteins of enterohemorrhagic and enteropathogenic *Escherichia coli* strains and their *ihf* and *ler* mutants. *Appl Environ Microbiol* **70:** (9) 5274.

Park YJ, Dixit A, Yoo JW, Bennetzen J. 2004. Further evidence of microcolinearity between barley and rice genomes at two orthologous regions. *Mol Cells* **17:** (3) 492.

Paterson AH, Bowers JE, Chapman BA. 2004. Ancient polyploidization predating divergence of the cereals, and its consequences for comparative genomics. *Proc Natl Acad Sci U S A* **101:** (26) 9903.

Rajashekara G, Glasner JD, Glover DA, Splitter GA. 2004. Comparative whole-genome hybridization reveals genomic islands in *Brucella* species. *J Bacteriol* **186:** (15) 5040.

Rodionov DA, Vitreschak AG, Mironov AA, Gelfand MS. 2004. Comparative genomics of the methionine metabolism in Gram-positive bacteria: A variety of regulatory systems. *Nucleic Acids Res* **32:** (11) 3340.

Samrakandi MM, Zhang C, Zhang M, Nietfeldt J, Kim J, Iwen PC, Olson ME, Fey PD, Duhamel GE, Hinrichs SH *et al*. 2004. Genome diversity among regional populations of *Francisella tularensis* subspecies *tularensis* and *Francisella tularensis* subspecies *holarctica* isolated from the US. *FEMS Microbiol Lett* **237:** (1) 9.

### 6 Pathways, gene families and regulons

Cochrane FC, Davin LB, Lewis NG. 2004. The *Arabidopsis* phenylalanine ammonia lyase gene family: Kinetic characterization of the flour PAL isoforms. *Phytochemistry* **65:** (11) 1557.

Tian CG, Wan P, Sun SH, Li JY, Chen MS. 2004. Genome-wide analysis of the GRAS gene family in rice and *Arabidopsis*. *Plant Mol Biol* **54:** (4) 519.

Wang LS, Trawick JD, Yamamoto R, Zamudio C. 2004. Genome-wide operon prediction in *Staphylococcus aureus*. *Nucleic Acids Res* **32:** (12) 3689.

Yang HH, Hu Y, Buetow KH, Lee MP. 2004. A computational approach to measuring coherence of gene expression in pathways. *Genomics* **84:** (1) 211.

Zelter A, Bencina M, Bowman BJ, Read ND. 2004. A comparative genomic analysis of the calcium signaling machinery in *Neurospora crassa, Magnaporthe grisea,* and *Saccharomyces cerevisiae*. *Fungal Genet Biol* **41:** (9) 827.

### 7 Pharmacogenomics

Abba MC, Drake JA, Hawkins KA, Hu YH, Sun HX, Notcovich C,

Copyright © 2005 John Wiley & Sons, Ltd.

Gaddis S, Sahin A, Baggerly K, Aldaz CM. 2004. Transcriptomic changes in human breast cancer progression as determined by serial analysis of gene expression. *Breast Cancer Res* **6:** (5) R499.

Aldred MA, Huang Y, Liyanarachchi S, Pellegata NS, Gimm O, Jhiang S, Davuluri RV, De la Chapelle A, Eng C. 2004. Papillary and follicular thyroid carcinomas show distinctly different microarray expression profiles and can be distinguished by a minimum of five genes. *J Clin Oncol* **22:** (17) 3531.

Allal AS, Kahne T, Reverdin AK, Lippert H, Schlegel W, Reymond MA. 2004. Radioresistance-related proteins in rectal cancer. *Proteomics* **4:** (8) 2261.

Allard L, Lescuyer P, Burgess J, Leung KY, Ward M, Walter N, Burkhard PR, Corthals G, Hochstrasser DF, Sanchez JC. 2004. ApoC-I and ApoC-III as potential plasmatic markers to distinguish between ischemic and hemorrhagic stroke. *Proteomics* **4:** (8) 2242.

Almeras L, Lefranc D, Drobecq H, De Seze J, Dubucquoi S, Vermersch P, Prin L. 2004. New antigenic candidates in multiple sclerosis: Identification by serological proteome analysis. *Proteomics* **4:** (7) 2184.

Almstrup K, Hoei-Hansen CE, Wirkner U, Blake J, Schwager C, Ansorge W, Nielsen JE, Skakkebaek NE, Meyts ERD, Leffers H. 2004. Embryonic stem cell-like features of testicular carcinoma *in situ* revealed by genome-wide gene expression profiling. *Cancer Res* **64:** (14) 4736.

Ashida S, Nakagawa H, Katagiri T, Furihata M, Iiizumi M, Anazawa Y, Tsunoda T, Takata R, Kasahara K, Miki T *et al*. 2004. Molecular features of the transition from prostatic intraepithelial neoplasia (PIN) to prostate cancer: Genome-wide gene-expression profiles of prostate cancers and PINs. *Cancer Res* **64:** (17) 5963.

Aston C, Jiang LX, Sokolov BP. 2004. Microarray analysis of postmortem temporal cortex from patients with schizophrenia. *J Neurosci Res* **77:** (6) 858.

Bai Y, Galetskiy D, Damoc E, Paschen C, Liu ZQ, Griese M, Liu SY, Przybylski M. 2004. High resolution mass spectrometric alveolar proteomics: Identification of surfactant protein SP-A and SP-D modifications in proteinosis and cystic fibrosis patients. *Proteomics* **4:** (8) 2300.

Bard MP, Hegmans JP, Hemmes A, Luider TM, Willemsen R, Severijnen LAA, Van Meerbeeck JP, Burgers SA, Hoogsteden HC, Lambrecht BN. 2004. Proteomic analysis of exosomes isolated from human malignant pleural effusions. *Am J Respir Cell Mol Biol* **31:** (1) 114.

Barker KS, Crisp S, Wiederhold N, Lewis RE, Bareither B, Eckstein J, Barbuch R, Bard M, Rogers PD. 2004. Genome-wide expression profiling reveals genes associated with amphotericin B and fluconazole resistance in experimentally induced antifungal resistant isolates of *Candida albicans*. *J Antimicrob Chemother* **54:** (2) 376.

Basso D, Millino C, Greco E, Romualdi C, Fogar P, Valerio A, Bellin M, Zambon CF, Navaglia F, Dussini N *et al*. 2004. Altered glucose metabolism and proteolysis in pancreatic cancer cell conditioned myoblasts: Searching for a gene expression pattern with a microarray analysis of 5000 skeletal muscle genes. *Gut* **53:** (8) 1159.

Blonder J, Terunuma A, Conrads TR, Chan KC, Yee C, Lucas DA, Schaefer CF, Yu LR, Issaq HJ, Veenstra TD *et al*. 2004. A proteomic characterization of the plasma membrane of human epidermis by high-throughput mass spectrometry. *J Investig Dermatol* **123:** (4) 691.

Booniakuakul JK, Syvanen M, Suryaprasad A, Bowlus CL, Solnick JV. 2004. Transcription profile of *Helicobacter pylori* in the human stomach reflects its physiology *in vivo*. *J Infect Dis* **190:** (5) 946.

Boshoff HIM, Myers TG, Copp BR, McNeil MR, Wilson MA, Barry CE. 2004. The transcriptional responses of *Mycobacterium tuberculosis* to inhibitors of metabolism - Novel insights into drug mechanisms of action. *J Biol Chem* **279:** (38) 40174.

Brennan C, Zhang YY, Leo C, Feng B, Cauwels C, Aguirre AJ, Kim MJ, Protopopov A, Chin L. 2004. High-resolution global profiling of genomic alterations with long oligonucleotide microarray. *Cancer Res* **64:** (14) 4744.

Brooks AR, Lelkes PI, Rubanyi GM. 2004. Gene expression profiling of vascular endothelial cells exposed to fluid mechanical forces: Relevance for focal susceptibility to atherosclerosis. *Endothelium* **11:** (1) 45.

Cacabelos R, Fernandez-Novoa L, Corzo L, Amado L, Pichel V, Lombardi V, Kubota Y. 2004. Phenotypic profiles and functional genomics in Alzheimer's disease and in dementia with a vascular component. *Neurol Res* **26:** (5) 459.

Cekaite L, Haug O, Myklebost O, Aldrin M, Ostenstad B, Holden M, Frigessi A, Hovig E, Sioud M. 2004. Analysis of the humoral immune response to immunoselected phage-displayed peptides by a microarray-based method. *Proteomics* **4:** (9) 2572.

Chen J, He QY, Yuen APW, Chiu JF. 2004. Proteomics of buccal squamous cell carcinoma: The involvement of multiple pathways in tumorigenesis. *Proteomics* **4:** (8) 2465.

Chung HW, Park SW, Chung JB, Kang JK, Kim JW, Kim HS, Hyung WJ, Noh SH, Song SY. 2004. Differences in genetic expression profiles between young-age and old-age gastric adenocarcinoma using cDNA microarray for endocrine disruptor study. *Oncol Rep* **12:** (1) 33.

Cicatiello L, Scafoglio C, Altucci L, Cancemi M, Natoli G, Facchiano A, Iazzetti G, Calogero R, Biglia N, De Bortoli M *et al*. 2004. A genomic view of estrogen actions in human breast cancer cells by expression profiling of the hormone-responsive transcriptome. *J Mol Endocrinol* **32:** (3) 719.

Clegg N, Abbott D, Ferguson C, Coleman R, Nelsons PS. 2004. Characterization and comparative analyses of transcriptomes from the normal and neoplastic human prostate. *Prostate* **60:** (3) 227.

Cutillas PR, Chalkley RJ, Hansen KC, Cramer R, Norden AGW, Waterfield MD, Burlingame AL, Unwin RJ. 2004. The urinary proteome in Fanconi syndrome implies specificity in the reabsorption of proteins by renal proximal tubule cells. *Am J Physiol* **287:** (3) F353.

Dahan S, Knutton S, Shaw RK, Crepin VF, Dougan G, Frankel G. 2004. Transcriptome of enterohemorrhagic *Escherichia coli* O157 adhering to eukaryotic plasma membranes. *Infect Immun* **72:** (9) 5452.

Dasu MRK, Barrow RE, Hawkins HK, McCauley RL. 2004. Gene expression profiles of giant hairy naevi. *J Clin Pathol* **57:** (8) 849.

Degauque N, Schadendorf D, Brouard S, Guillet M, Sebille F, Hohn H, Pallier A, Ruiz C, Dupont A, Chapin S *et al*. 2004. Blood T-cell Vβ transcriptome in melanoma patients. *Int J Cancer* **110:** (5) 721.

Demir AY, Demol H, Puype M, De Goeij AFPM, Dunselman GAJ, Herrler A, Evers JLH, Vandekerckhove J, Groothuis PG. 2004. Proteome analysis of human mesothelial cells during epithelial to mesenchymal transitions induced by shed menstrual effluent. *Proteomics* **4:** (9) 2608.

Dumont D, Noben JP, Raus J, Stinissen P, Robben J. 2004. Proteomic analysis of cerebrospinal fluid from multiple sclerosis patients. *Proteomics* **4:** (7) 2117.

Durr E, Yu JY, Krasinska KM, Carver LA, Yates JR, Testa JE, Oh P, Schnitzer JE. 2004. Direct proteomic mapping of the lung microvascular endothelial cell surface *in vivo* and in cell culture. *Nat Biotechnol* **22:** (8) 985.

Flechner SM, Kurian SM, Head SR, Sharp SM, Whisenant TC, Zhang J, Chismar JD, Horvath S, Mondala T, Gilmartin T *et al*. 2004. Kidney transplant rejection and tissue injury by gene profiling of biopsies and peripheral blood lymphocytes. *Am J Transplant* **4:** (9) 1475.

Fowler LJ, Lovell MO, Izbicka E. 2004. Fine-needle aspiration in PreservCyt®: A novel and reproducible method for possible ancillary proteomic pattern expression of breast neoplasms by SELDI-TOF. *Mod Pathol* **17:** (8) 1012.

Frazier MC, Jackson KM, Jankowska-Stephens E, Anderson MG, Harris WB. 2004. Proteomic analysis of proteins altered by dibenzoylmethane in human prostatic cancer LNCaP cells. *Proteomics* **4:** (9) 2814.

Frisk A, Schurr JR, Wang GS, Bertucci DC, Marrero L, Hwang SH, Hassett DJ, Schurr MJ. 2004. Transcriptome analysis of *Pseudomonas aeruginosa* after interaction with human airway epithelial cells. *Infect Immun* **72:** (9) 5433.

Fujimoto N, Igarashi K, Kanno J, Honda H, Inoue T. 2004. Identification of estrogen-responsive genes in the GH3 cell line by cDNA microarray analysis. *J Steroid Biochem Mol Biol* **91:** (3) 121.

Fujiwara K, Ochiai M, Ohta T, Ohki M, Aburatani H, Nagao M, Sugimura T, Nakagama H. 2004. Global gene expression analysis of rat colon cancers induced by a food-borne carcinogen, 2-amino-1-methyl-6-phenylimidazo[4,5-*b*]pyridine. *Carcinogenesis* **25:** (8) 1495.

Gianazza E, Allegra L, Bucchioni E, Eberini I, Pughsi L, Blasi F, Terzano C, Wait R, Sirtori CR. 2004. Increased keratin content detected by proteomic analysis of exhaled breath condensate from healthy persons who smoke. *Am J Med* **117:** (1) 51.

Gravett MG, Novy MJ, Rosenfeld RG, Reddy AP, Jacob T, Turner M, McCormack A, Lapidus JA, Hitti J, Eschenbach DA *et al*. 2004. Diagnosis of intra-amniotic infection by proteomic profiling and identification of novel biomarkers. *JAMA* **292:** (4) 462.

Gretzer MB, Chan DW, Van Rootselaar CL, Rosenzweig JM, Dalrymple S, Mangold LA, Partin AW, Veltri RW. 2004. Proteomic analysis of

Copyright © 2005 John Wiley & Sons, Ltd. 

Dunning prostate cancer cell lines with variable metastatic potential using SELDI-TOF. *Prostate* **60:** (4) 325.
Grill C, Gheyas F, Dayananth P, Jin WH, Ding W, Qiu P, Wang LQ, Doll RJ, English JM. 2004. Analysis of the ERK1,2 transcriptome in mammary epithelial cells. *Biochem J* **381:** (3) 635.
Haagerup A, Bjerke T, Schiotz PO, Dahl R, Binderup HG, Tan QH, Kruse TA. 2004. Atopic dermatitis - A total genome-scan for susceptibility genes. *Acta Derm Venereol* **84:** (5) 346.
Hall JL, Grindle S, Han XQ, Fermin D, Park S, Chen YJ, Bache RJ, Mariash A, Guan ZJ, Ormaza S *et al*. 2004. Genomic profiling of the human heart before and after mechanical support with a ventricular assist device reveals alterations in vascular signaling networks. *Physiol Genomics* **17:** (3) 283.
Harris MN, Ozpolat B, Abdi F, Gu S, Legler A, Mawuenyega KG, Tirado-Gomez M, Lopez-Berestein G, Chen X. 2004. Comparative proteomic analysis of all-*trans*-retinoic acid treatment reveals systematic posttranscriptional control mechanisms in acute promyelocytic leukemia. *Blood* **104:** (5) 1314.
Hart GT, Shaffer DJ, Akilesh S, Brown AC, Moran L, Roopenian DC, Baker PJ. 2004. Quantitative gene expression profiling implicates genes for susceptibility and resistance to alveolar bone loss. *Infect Immun* **72:** (8) 4471.
Hathout Y, Gehrmann ML, Chertov A, Fenselau C. 2004. Proteomic phenotyping: Metastatic and invasive breast cancer. *Cancer Lett* **210:** (2) 245.
Hauser P, Schwarz C, Mitterbauer C, Regele HM, Muhlbacher F, Mayer G, Perco P, Mayer B, Meyer TW, Oberbauer R. 2004. Genome-wide gene-expression patterns of donor kidney biopsies distinguish primary allograft function. *Lab Invest* **84:** (3) 353.
Heinloth AN, Irwin RD, Boorman GA, Nettesheim P, Fannin RD, Sieber SO, Snell ML, Tucker CJ, Li LP, Travlos GS *et al*. 2004. Gene expression profiling of rat livers reveals indicators of potential adverse effects. *Toxicol Sci* **80:** (1) 193.
Hellman K, Alaiya AA, Schedvins K, Steinberg W, Hellstrom AC, Auer G. 2004. Protein expression patterns in primary carcinoma of the vagina. *Br J Cancer* **91:** (2) 319.
Herzog A, Kindermann B, Doring F, Daniel H, Wenzel U. 2004. Pleiotropic molecular effects of the pro-apoptotic dietary constituent flavone in human colon cancer cells identified by protein and mRNA expression profiling. *Proteomics* **4:** (8) 2455.
Hildebrandt HAT, Salavaggione OE, Martin YN, Flynn HC, Jalal S, Wieben ED, Weinshilboum RM. 2004. Human *SULT1A3* pharmacogenetics: Gene duplication and functional genomic studies. *Biochem Biophys Res Commun* **321:** (4) 870.
Holland CM, Saidi SA, Evans AL, Sharkey AM, Latimer JA, Crawford RAF, Charnock-Jones DS, Print CG, Smith SK. 2004. Transcriptome analysis of endometrial cancer identifies peroxisome proliferator-activated receptors as potential therapeutic targets. *Mol Cancer Ther* **3:** (8) 993.
Holleman A, Cheok MH, Den Boer ML, Yang WJ, Veerman AJP, Kazemier KM, Pei DQ, Cheng C, Pui CH, Relling MV *et al*. 2004. Gene-expression patterns in drug-resistant acute lymphoblastic leukemia cells and response to treatment. *N Engl J Med* **351:** (6) 533.
Holtkamp N, Reuss DE, Atallah I, Kuban RJ, Hartmann C, Mautner VF, Frahm S, Friedrich RE, Algermissen B, Pham VA *et al*. 2004. Subclassification of nerve sheath tumors by gene expression profiling. *Brain Pathol* **14:** (3) 258.
Hooshdaran MZ, Barker KS, Hilliard GM, Kusch H, Morschhauser J, Rogers PD. 2004. Proteomic analysis of azole resistance in *Candida albicans* clinical isolates. *Antimicrob Agents Chemother* **48:** (7) 2733.
Huff JL, Hansen LM, Solnick JV. 2004. Gastric transcription profile of *Helicobacter pylori* infection in the rhesus macaque. *Infect Immun* **72:** (9) 5216.
Hutter B, Schaab C, Albrecht S, Borgmann M, Brunner NA, Freiberg C, Ziegelbauer K, Rock CO, Ivanov I, Loferer H. 2004. Prediction of mechanisms of action of antibacterial compounds by gene expression profiling. *Antimicrob Agents Chemother* **48:** (8) 2838.
Iwafuchi H, Mori N, Takahashi T, Yatabe Y. 2004. Phenotypic composition of salivary gland tumors: An application analysis to tissue of principle component microarray data. *Mod Pathol* **17:** (7) 803.
Jin M, Opalek JM, Marsh CB, Wu HFM. 2004. Proteome comparison of alveolar macrophages with monocytes reveals distinct protein characteristics. *Am J Respir Cell Mol Biol* **31:** (3) 322.
Jinawath N, Furukawa Y, Hasegawa S, Li MH, Tsunoda T, Satoh S, Yamaguchi T, Imamura H, Inoue M, Shiozaki H *et al*. 2004. Comparison of gene-expression profiles between diffuse- and intestinal-type gastric cancers using a genome-wide cDNA microarray. *Oncogene* **23:** (40) 6830.
Juan HF, Chen JH, Hsu WT, Huang SC, Chen ST, Lin JYC, Chang YW, Chiang CY, Wen LL, Chan DC *et al*. 2004. Identification of tumor-associated plasma biomarkers using proteomic techniques: From mouse to human. *Proteomics* **4:** (9) 2766.
Jugdutt BI, Sawicki G. 2004. $AT^1$receptor blockade alters metabolic, functional and structural proteins after reperfused myocardial infarction: Detection using proteomics. *Mol Cell Biochem* **263:** (1) 179.
Jura J, Wegrzyn P, Zarebski A, Wladyka B, Koj A. 2004. Identification of changes in the transcriptome profile of human hepatoma HePG2 cells stimulated with interleukin-1 β. *Biochim Biophys Acta* **1689:** (2) 120.
Kaiser T, Kamal H, Rank A, Kolb HJ, Holler E, Ganser A, Hertenstein B, Mischak H, Weissinger EM. 2004. Proteomics applied to the clinical follow-up of patients after allogeneic hematopoietic stem cell transplantation. *Blood* **104:** (2) 340.
Karababa M, Coste AT, Rognon B, Bille J, Sanglard D. 2004. Comparison of gene expression profiles of *Candida albicans* azole-resistant clinical isolates and laboratory strains exposed to drugs inducing multidrug transporters. *Antimicrob Agents Chemother* **48:** (8) 3064.
Kettunen E, Vivo C, Gattacceca F, Knuutila S, Jaurand MC. 2004. Gene expression profiles in human mesothelioma cell lines in response to interferon-γ treatment. *Cancer Genet Cytogenet* **152:** (1) 42.
Kim JL, Jung HH. 2004. Proteomic analysis of cholesteatoma. *Acta Otolaryngol (Stockh)* **124:** (7) 783.
Kim SY, Chudapongse N, Lee SM, Levin MC, Oh JT, Park HJ, Ho IK. 2004. Proteomic analysis of phosphotyrosyl proteins in the rat brain: Effect of butorphanol dependence. *J Neurosci Res* **77:** (6) 867.
Kluger HM, Kluger Y, Gilmore-Hebert M, DiVito K, Chang JT, Rodov S, Mironenko O, Kacinski BM, Perkins AS, Sapi E. 2004. cDNA microarray analysis of invasive and tumorigenic phenotypes in a breast cancer model. *Lab Invest* **84:** (3) 320.
Kreunin P, Urquidi V, Lubman DM, Goodison S. 2004. Identification of metastasis-associated proteins in a human tumor metastasis model using the mass-mapping technique. *Proteomics* **4:** (9) 2754.
Krieg RC, Knuechel R, Schiffmann E, Liotta LA, Petricoin EF, Herrmann PC. 2004. Mitochondrial proteome: Cancer-altered metabolism associated with cytochrome *c* oxidase subunit level variation. *Proteomics* **4:** (9) 2789.
Kusch H, Biswas K, Schwanfelder S, Engelmann S, Rogers PD, Hecker M, Morschhauser J. 2004. A proteomic approach to understanding the development of multidrug-resistant *Candida albicans* strains. *Mol Genet Genomics* **271:** (5) 554.
Lal S, Lui R, Nguyen L, Macdonald P, Denyer G, Dos Remedios C. 2004. Increases in leukocyte cluster of differentiation antigen expression during cardiopulmonary bypass in patients undergoing heart transplantation. *Proteomics* **4:** (7) 1918.
Leclerc N, Luppen CA, Ho VV, Nagpal S, Hacia JG, Smith E, Frenkel B. 2004. Gene expression profiling of glucocorticoid-inhibited osteoblasts. *J Mol Endocrinol* **33:** (1) 175.
Lee JS, Chu IS, Heo J, Calvisi DF, Sun ZT, Roskams T, Durnez A, Demetris AJ, Thorgeirsson SS. 2004. Classification and prediction of survival in hepatocellular carcinoma by gene expression profiling. *Hepatology* **40:** (3) 667.
Lee MS, Hanspers K, Barker CS, Korn AP, McCune JM. 2004. Gene expression profiles during human $CD4^+$ T cell differentiation. *Int Immunol* **16:** (8) 1109.
Leigh JA, Ward PN, Field TR. 2004. The exploitation of the genome in the search for determinants of virulence in *Streptococcus uberis*. *Vet Immunol Immunopathol* **100:** (3-4) 145.
Lescuyer P, Allard J, Zimmermann-Ivol CG, Burgess JA, Hughes-Frutiger S, Burkhard PR, Sanchez JC, Hochstrasser DF. 2004. Identification of post-mortem cerebrospinal fluid proteins as potential biomarkers of ischemia and neurodegeneration. *Proteomics* **4:** (8) 2234.
Li XM, Patel BB, Blagoi EL, Patterson MD, Seehozer SH, Zhang T, Damle S, Gao ZQ, Boman B, Yeung AT. 2004. Analyzing alkaline proteins in human colon crypt proteome. *J Proteome Res* **3:** (4) 821.
Liu Y, Chen Q, Zhang JT. 2004. Tumor suppressor gene 14-3-3σ is down-regulated whereas the proto-oncogene translation elongation factor 1δ is up-regulated in non-small cell lung cancers as identified by

Copyright © 2005 John Wiley & Sons, Ltd. 

proteomic profiling. *J Proteome Res* **3:** (4) 728.
Lopez-Bigas N, Ouzounis CA. 2004. Genome-wide identification of genes likely to be involved in human genetic disease. *Nucleic Acids Res* **32:** (10) 3108.
Lu XCM, Williams AJ, Yao C, Berti R, Hartings JA, Whipple R, Vahey MT, Polavarapu RG, Woller KL, Tortella FC *et al*. 2004. Microarray analysis of acute and delayed gene expression profile in rats after focal ischemic brain injury and reperfusion. *J Neurosci Res* **77:** (6) 843.
Lum AM, Huang JQ, Hutchinson CR, Kao CM. 2004. Reverse engineering of industrial pharmaceutical-producing actinomycete strains using DNA microarrays. *Metab Eng* **6:** (3) 186.
Luyendyk JP, Mattes WB, Burgoon LD, Zacharewski TR, Maddox JF, Cosma GN, Ganey PE, Roth RA. 2004. Gene expression analysis points to hemostasis in livers of rats cotreated with lipopolysaccharide and ranitidine. *Toxicol Sci* **80:** (1) 203.
Maccarrone G, Milfay D, Birg I, Rosenhagen M, Holsboer F, Grimm R, Bailey J, Zolotarjova N, Turck CW. 2004. Mining the human cerebrospinal fluid proteome by immunodepletion and shotgun mass spectrometry. *Electrophoresis* **25:** (14) 2402.
Magnani A, Barbucci R, Lamponi S, Chiumento A, Paffetti A, Trabalzini L, Martelli P, Santucci A. 2004. Two-step elution of human serum proteins from different glass modified bioactive surfaces: A comparative proteomic analysis of adsorption patterns. *Electrophoresis* **25:** (14) 2413.
Marengo E, Robotti E, Righetti PG, Campostrini N, Pascali J, Ponzoni M, Hamdan M, Astner H. 2004. Study of proteomic changes associated with healthy and tumoral murine samples in neuroblastoma by principal component analysis and classification methods. *Clin Chim Acta* **345:** (1-2) 55.
Matsuzaki S, Canis M, Vaurs-Barriere C, Pouly JL, Boespflug-Tanguy O, Penault-Llorca F, Dechelotte P, Dastugue B, Okamura K, Mage G. 2004. DNA microarray analysis of gene expression profiles in deep endometriosis using laser capture microdissection. *Mol Hum Reprod* **10:** (10) 719.
Messana I, Cabras T, Inzitari R, Lupi A, Zuppi C, Olmi C, Fadda MB, Cordaro M, Giardina B, Castagnola M. 2004. Characterization of the human salivary basic proline-rich protein complex by a proteomic approach. *J Proteome Res* **3:** (4) 792.
Miller DV, Leontovich AA, Lingle WL, Suman VJ, Mertens ML, Lillie J, Ingalls KA, Perez EA, Ingle JN, Couch FJ *et al*. 2004. Utilizing Nottingham Prognostic Index in microarray gene expression profiling of breast carcinomas. *Mod Pathol* **17:** (7) 756.
Monticone M, Liu Y, Tonachini L, Mastrogiacomo M, Parodi S, Quarto R, Cancedda R, Castagnola P. 2004. Gene expression profile of human bone marrow stromal cells determined by restriction fragment differential display analysis. *J Cell Biochem* **92:** (4) 733.
Moore JMR, Galicia SJ, McReynolds AC, Nguyen NH, Scanlan TS, Guy RK. 2004. Quantitative proteomics of the thyroid hormone receptor-coregulator interactions. *J Biol Chem* **279:** (26) 27584.
Mukhopadhyay S, Miller RD, Summersgill JT. 2004. Analysis of altered protein expression patterns of *Chlamydia pneumoniae* by an integrated, proteome-works system. *J Proteome Res* **3:** (4) 878.
Muller-Tidow C, Diederichs S, Thomas M, Serve H. 2004. Genome-wide screening for prognosis-predicting genes in early-stage non-small-cell lung cancer. *Lung Cancer* **45:** (S2) S145.
Mycko MP, Papoian R, Boschert U, Raine CS, Selmaj KW. 2004. Microarray gene expression profiling of chronic active and inactive lesions in multiple sclerosis. *Clin Neurol Neurosurg* **106:** (3) 223.
Ng RK, Lau CYL, Lee SMY, Tsui SKW, Fung KP, Waye MMY. 2004. cDNA microarray analysis of early gene expression profiles associated with hepatitis B virus X protein-mediated hepatocarcinogenesis. *Biochem Biophys Res Commun* **322:** (3) 827.
Nilsson S, Ramstrom M, Palmblad M, Axelsson D, Bergquist J. 2004. Explorative study of the protein composition of amniotic fluid by liquid chromatography electrospray ionization Fourier transform ion cyclotron resonance mass spectrometry. *J Proteome Res* **3:** (4) 884.
Ohki-Kaneda R, Ohashi J, Yamamoto K, Ueno S, Ota J, Choi YL, Koinuma K, Yamashita Y, Misawa Y, Fuse K *et al*. 2004. Cardiac function-related gene expression profiles in human atrial myocytes. *Biochem Biophys Res Commun* **320:** (4) 1328.
Ohki R, Yamamoto K, Ueno S, Mano H, Misawa Y, Fuse K, Ikeda U, Shimada K. 2004. Transcriptional profile of genes induced in human atrial myocardium with pressure overload. *Int J Cardiol* **96:** (3) 381.
Ong ES, Len SM, Lee ACH, Chui P, Chooi KF. 2004. Proteomic analysis of mouse liver for the evaluation of effects of Scutellariae Radix by liquid chromatography with tandem mass spectrometry. *Rapid Commun Mass Spectrom* **18:** (21) 2522.
Ornstein DK, Rayford W, Fusaro VA, Conrads TP, Ross SJ, Hitt BA, Wiggins WW, Veenstra TD, Liotta LA, Petricoin EF. 2004. Serum proteomic profiling can discriminate prostate cancer from benign prostates in men with total prostate specific antigen levels between 2.5 and 15.0 ng/ml. *J Urol* **172:** (4 Pt 1) 1302.
Palotas A, Puskas LG, Kitajka K, Palotas M, Molnar J, Pakaski M, Janka Z, Penke B, Kalman J. 2004. Altered response to mirtazapine on gene expression profile of lymphocytes from Alzheimer's patients. *Eur J Pharmacol* **497:** (3) 247.
Pan JZ, Jornsten R, Hart RP. 2004. Screening anti-inflammatory compounds in injured spinal cord with microarrays: A comparison of bioinformatics analysis approaches. *Physiol Genomics* **17:** (2) 201.
Pan TL, Wang PW, Huang CC, Goto S, Chen CL. 2004. Expression, by functional proteomics, of spontaneous tolerance in rat orthotopic liver transplantation. *Immunology* **113:** (1) 57.
Parodi-Talice A, Duran R, Arrambide N, Prieto V, Pineyro MD, Pritsch O, Cayota A, Cervenansky C, Robello C. 2004. Proteome analysis of the causative agent of Chagas disease: *Trypanosoma cruzi*. *Int J Parasitol* **34:** (8) 881.
Petricoin EF, Liotta LA. 2004. Proteomic pattern complexity reveals a rich and uncharted continent of biomarkers (Letter). *Clin Chem* **50:** (8) 1476.
Pisitkun T, Shen RF, Knepper MA. 2004. Identification and proteomic profiling of exosomes in human urine. *Proc Natl Acad Sci U S A* **101:** (36) 13368.
Poon TCW, Chan KCA, Ng PC, Chiu RWK, Ang IL, Tong YK, Ng EKO, Cheng FWT, Li AM, Hon EKL *et al*. 2004. Serial analysis of plasma proteomic signatures in pediatric patients with severe acute respiratory syndrome and correlation with viral load. *Clin Chem* **50:** (8) 1452.
Price RM, Tulsyan N, Dermody JJ, Schwalb M, Soteropoulos P, Castronuovo JJ. 2004. Gene expression after crush injury of human saphenous vein: Using microarrays to define the transcriptional profile. *J Am Coll Surg* **199:** (3) 411.
Qiu TH, Chandramouli GVR, Hunter KW, Alkharouf NW, Green JE, Liu ET. 2004. Global expression profiling identifies signatures of tumor virulence in MMTV-PyMT-transgenic mice: Correlation to human disease. *Cancer Res* **64:** (17) 5973.
Rhodes DR, Yu JJ, Shanker K, Deshpande N, Varambally R, Ghosh D, Barrette T, Pandey A, Chinnaiyan AM. 2004. Large-scale meta-analysis of cancer microarray data identifies common transcriptional profiles of neoplastic transformation and progression. *Proc Natl Acad Sci U S A* **101:** (25) 9309.
Ricciarelli R, D'Abramo C, Massone S, Marinari UM, Pronzato MA, Tabaton M. 2004. Microarray analysis in Alzheimer's disease and normal aging. *IUBMB Life* **56:** (6) 349.
Robson B, Mushlin R. 2004. Clinical and pharmacogenomic data mining: 2. A simple method for the combination of information form associations and multivariances to facilitate analysis, decision, and design in clinical research and practice. *J Proteome Res* **3:** (4) 697.
Rudnicki M, Eder S, Schratzberger G, Mayer B, Meyer T, Tonko M, Mayer G. 2004. Reliability of T7-based mRNA linear amplification validated by gene expression analysis of human kidney cells using cDNA microarrays. *Nephron Exp Nephrol* **97:** (3) 86.
Sadlier DM, Connolly SB, Kieran NE, Roxburgh S, Brazil DP, Kairaitis L, Wang Y, Harris DCH, Doran P, Brady HR. 2004. Sequential extracellular matrix-focused and baited-global cluster analysis of serial transcriptomic profiles identifies candidate modulators of renal tubulointerstitial fibrosis in murine adriamycin-induced nephropathy. *J Biol Chem* **279:** (28) 29670.
Saito M, Szakall I, Toth R, Kovacs KM, Oros M, Prasad VVTS, Blumenberg M, Vadasz C. 2004. Mouse striatal transcriptome analysis: Effects of oral self-administration of alcohol. *Alcohol* **32:** (3) 223.
Sanchez JC, Guillaume E, Lescuyer P, Allard L, Carrette O, Scherl A, Burgess J, Corthals GL, Burkhard PR, Hochstrasser DF. 2004. Cystatin C as a potential cerebrospinal fluid marker for the diagnosis of Creutzfeldt-Jakob disease. *Proteomics* **4:** (8) 2229.
Sarto C, Valsecchi C, Magni F, Tremolada L, Arizzi C, Cordani N, Casellato S, Doro G, Favini P, Perego RA *et al*. 2004. Expression of heat shock protein 27 in human renal cell carcinoma. *Proteomics* **4:** (8) 2252.
Seike M, Kondo T, Fujii K, Yamada T, Gemma A, Kudoh S, Hirohashi

Copyright © 2005 John Wiley & Sons, Ltd. 

S. 2004. Proteomic signature of human cancer cells. *Proteomics* **4:** (9) 2776.
Shipkova M, Beck H, Voland A, Armstrong VW, Grone HJ, Oellerich M, Wieland E. 2004. Identification of protein targets for mycophenolic acid acyl glucuronide in rat liver and colon tissue. *Proteomics* **4:** (9) 2728.
Signor L, Tigani B, Beckmann N, Falchetto R, Stoeckli M. 2004. Two-dimensional electrophoresis protein profiling and identification in rat bronchoalveolar lavage fluid following allergen and endotoxin challenge. *Proteomics* **4:** (7) 2101.
Smith SD, She YM, Roberts EA, Sarkar B. 2004. Using immobilized metal affinity chromatography, two-dimensional electrophoresis and mass spectrometry to identify hepatocellular proteins with copper-binding ability. *J Proteome Res* **3:** (4) 834.
Sogayar MC, Camargo AA. 2004. A transcript finishing initiative for closing gaps in the human transcriptome. *Genome Res* **14:** (7) 1413.
Soltys SG, Le QT, Shi GY, Tibshirani R, Giaccia AJ, Koong AC. 2004. The use of plasma surface-enhanced laser desorption/ionization time-of-flight mass spectrometry proteomic patterns for detection of head and neck squamous cell cancers. *Clin Cancer Res* **10:** (14) 4806.
Spira A, Beane J, Shah V, Liu G, Schembri F, Yang XM, Palma J, Brody JS. 2004. Effects of cigarette smoke on the human airway epithelial cell transcriptome. *Proc Natl Acad Sci U S A* **101:** (27) 10143.
Sriuranpong V, Mutirangura A, Gillespie JW, Patel V, Amornphimoltham P, Molinolo AA, Kerekhanjanarong V, Supanakorn S, Supiyaphun P, Rangdaeng S *et al*. 2004. Global gene expression profile of nasopharyngeal carcinoma by laser capture microdissection and complementary DNA microarrays. *Clin Cancer Res* **10:** (15) 4944.
Steiner G, Suter L, Boess F, Gasser R, De Vera MC, Albertini S, Ruepp S. 2004. Discriminating different classes of toxicants by transcript profiling. *Environ Health Perspect* **112:** (12) 1236.
Sung HJ, Kim YS, Kim IS, Jang SW, Kim YR, Na DS, Han KH, Hwang BG, Park DS, Ko JS. 2004. Proteomic analysis of differential protein expression in neuropathic pain and electroacupuncture treatment models. *Proteomics* **4:** (9) 2805.
Tagawa H, Tsuzuki S, Suzuki R, Karnan S, Ota A, Kameoka Y, Suguro M, Matsuo K, Yamaguchi M, Okamoto M *et al*. 2004. Genome-wide array-based comparative genomic hybridization of diffuse large B-cell lymphoma: Comparison between CD5-positive and CD5-negative cases. *Cancer Res* **64:** (17) 5948.
Tan YX, Shi LB, Tong WD, Hwang GTG, Wang C. 2004. Multi-class tumor classification by discriminant partial least squares using microarray gene expression data and assessment of classification models. *Comput Biol Chem* **28:** (3) 235.
Teuffel O, Dettling M, Cario G, Stanulla M, Schrappe M, Buhlmann P, Niggl FK, Schafer BW. 2004. Gene expression profiles and risk stratification in childhood acute lymphoblastic leukemia. *Haematologica* **89:** (7) 801.
Ueda HR, Chen WB, Minami Y, Honma S, Honma K, Iino M, Hashimoto S. 2004. Molecular-timetable methods for detection of body time and rhythm disorders from single-time-point genome-wide expression profiles. *Proc Natl Acad Sci U S A* **101:** (31) 11227.
Ullmann R, Morbini P, Halbwedl I, Bongiovanni M, Gogg-Kammerer M, Papotti M, Gabor S, Renner H, Popper HH. 2004. Protein expression profiles in adenocarcinomas and squamous cell carcinomas of the lung generated using tissue microarrays. *J Pathol* **203:** (3) 798.
Van Delft JHM, Van Agen E, Van Breda SGJ, Herwijnen MH, Staal YCM, Kleinjans JCS. 2004. Discrimination of genotoxic from non-genotoxic carcinogens by gene expression profiling. *Carcinogenesis* **25:** (7) 1265.
Vanhentenrijk V, De Wolf-Peeters C, Wlodarska I. 2004. Comparative expressed sequence hybridization studies of hairy cell leukemia show uniform expression profile and imprint of spleen signature. *Blood* **104:** (1) 250.
Vemula M, Berthiaume F, Jayaraman A, Yarmush ML. 2004. Expression profiling analysis of the metabolic and inflammatory changes following burn injury in rats. *Physiol Genomics* **18:** (1) 87.
Vlahou A, Giannopoulos A, Gregory BW, Manousakas T, Kondylis FI, Wilson LL, Schelhammer PF, Wright GL, Semmes OJ. 2004. Protein profiling in urine for the diagnosis of bladder cancer. *Clin Chem* **50:** (8) 1438.
Wang DJ, Jensen RH, Williams KE, Pallavicini MG. 2004. Differential protein expression in MCF7 breast cancer cells transfected with ErbB2, neomycin resistance and luciferase plus yellow fluorescent protein. *Proteomics* **4:** (7) 2175.
Wang HX, Kachman MT, Schwartz DR, Cho KR, Lubman DM. 2004. Comprehensive proteome analysis of ovarian cancers using liquid phase separation, mass mapping and tandem mass spectrometry: A strategy for identification of candidate cancer biomarkers. *Proteomics* **4:** (8) 2476.
Wang XC, Xu SY, Wu X, Song HD, Mao YF, Fan HY, Yu F, Mou B, Gu YY, Xu LQ *et al*. 2004. Gene expression profiling in human insulinoma tissue: Genes involved in the insulin secretion pathway and cloning of novel full-length cDNAs. *Endocr Relat Cancer* **11:** (2) 295.
Wang YT, Huang ZJ, Chang HM. 2004. Proteomic analysis of human leukemic U937 cells incubated with conditioned medium of mononuclear cells stimulated by proteins from dietary mushroom of *Agrocybe aegerita*. *J Proteome Res* **3:** (4) 890.
Wenzel U, Herzog A, Kuntz S, Daniel H. 2004. Protein expression profiling identifies molecular targets of quercetin as a major dietary flavonoid in human colon cancer cells. *Proteomics* **4:** (7) 2160.
Wijnands MVW, Van Erk MJ, Doornbos RP, Krul CAM, Woutersen RA. 2004. Do aberrant crypt foci have predictive value for the occurrence of colorectal tumours? Potential of gene expression profiling in tumours. *Food Chem Toxicol* **42:** (10) 1629.
Williams RD, Hing SN, Greer BT, Whiteford CC, Wei JS, Natrajan R, Kelsey A, Rogers S, Campbell C, Pritchard-Jones K *et al*. 2004. Prognostic classification of relapsing favorable histology Wilms tumor using cDNA microarray expression profiling and support vector machines. *Gene Chromosomes Cancer* **41:** (1) 65.
Wilson RA, Curwen RS, Braschi S, Hall SL, Coulson PS, Ashton PD. 2004. From genomes to vaccines *via* the proteome. *Mem Inst Oswaldo Cruz* **99:** (5) 45.
Wong YF, Cheung TH, Lo KWK, Wang VW, Chan CS, Ng TB, Chung TKH, Mok SC. 2004. Protein profiling of cervical cancer by protein-biochips: Proteomic scoring to discriminate cervical cancer from normal cervix. *Cancer Lett* **211:** (2) 227.
Xiao XY, Zhao XD, Liu JK, Guo FZ, Liu DH, He DC. 2004. Discovery of laryngeal carcinoma by serum proteomic pattern analysis. *Sci China C Life Sci* **47:** (3) 219.
Xu Y, Ramos EJB, Middleton F, Romanova I, Quinn R, Chen C, Das U, Inui A, Meguid MM. 2004. Gene expression profiles post Roux-en-Y gastric bypass. *Surgery* **136:** (2) 246.
Yokoo H, Kondo T, Fujii K, Yamada T, Todo S, Hirohashi S. 2004. Proteomic signature corresponding to α fetoprotein expression in liver cancer cells. *Hepatology* **40:** (3) 609.
Yokoyama Y, Kuramitsu Y, Takashima M, Lizuka N, Toda T, Terai S, Sakaida I, Oka M, Nakamura K, Okita K. 2004. Proteomic profiling of proteins decreased in hepatocellular carcinoma from patients infected with hepatitis C virus. *Proteomics* **4:** (7) 2111.
Young T, Mei F, Yang G, Thompson-Lanza J, Liu J, Cheng X. 2004. Activation of antioxidant pathways in Ras-mediated oncogenic transformation of human surface ovarian epithelial cells revealed by functional proteomics and mass spectrometry. *Cancer Res* **64:** (3) 4577.
Yu M, Chen DM, Hu G, Wang H. 2004. Proteomic response analysis of endothelial cells of human coronary artery to stimulation with carbachol. *Acta Pharmacol Sin* **25:** (9) 1124.
Zhang JB, Li QG, Nguyen TD, Tremblay TL, Stone E, To R, Kelly J, MacKenzie CR. 2004. A pentavalent single-domain antibody approach to tumor antigen discovery and the development of novel proteomics reagents. *J Mol Biol* **341:** (1) 161.
Zhang Z, Bast RC, Yu YH, Li JN, Sokoll LJ, Rai AJ, Rosenzweig JM, Cameron B, Wang YY, Meng XY *et al*. 2004. Three biomarkers identified from serum proteomic analysis for the detection of early stage ovarian cancer. *Cancer Res* **64:** (16) 5882.
Zhou DS, Han YP, Dai EH, Song YJ, Pei DC, Zhai JH, Du ZM, Wang J, Guo ZB, Yang RF. 2004. Defining the genome content of live plague vaccines by use of whole-genome DNA microarray. *Vaccine* **22:** (25-26) 3367.
Zhuang Z, Lee YS, Zeng W, Furuta M, Valyi-Nagy T, Johnson MD, Vnencak-Jones CL, Woltjer RL, Weil RJ. 2004. Molecular genetic and proteomic analysis of synchronous malignant gliomas. *Neurology* **62:** (12) 2316.

### 8 EST, cDNA and other clone resources

Aguero F, Ben Abdellah K, Tekiel V, Sanchez DO, Gonzalez A. 2004.

Copyright © 2005 John Wiley & Sons, Ltd. 

Generation and analysis of expressed sequence tags from *Trypanosoma cruzi* trypomastigote and amastigote cDNA libraries. *Mol Biochem Parasitol* **136:** (2) 221.
Asamizu E, Nakamura Y, Sato S, Tabata S. 2004. Characteristics of the *Lotus japonicus* gene repertoire deduced from large-scale expressed sequence tag (EST) analysis. *Plant Mol Biol* **54:** (3) 405.
Casey OM, Fitzpatrick R, McInerney JO, Morris DG, Powell R, Sreenan JM. 2004. Analysis of gene expression in the bovine corpus luteum through generation and characterisation of 960 ESTs. *Biochim Biophys Acta* **1679:** (1) 10.
Fei ZJ, Tang X, Alba RM, White JA, Ronning CM, Martin GB, Tanksley SD, Giovannoni JJ. 2004. Comprehensive EST analysis of tomato and comparative genomics of fruit ripening. *Plant J* **40:** (1) 47.
Hollich V, Johnson E, Furlong EE, Beckmann B, Carlson J, Celniker SE, Hoheisel JD. 2004. Creation of a minimal tiling path of genomic clones for *Drosophila*: Provision of a common resource. *Biotechniques* **37:** (2) 282.
Ida H, Boylan SA, Weigel AL, Smit-McBride Z, Chao A, Gao J, Buchoff P, Wistow G, Hjelmeland LM. 2004. EST analysis of mouse retina and RPE/choroid cDNA libraries. *Mol Vis* **10:** (55) 439.
Krzywinski M, Bosdet I, Smaillus D, Chiu R, Mathewson C, Wye N, Barber S, Brown-John M, Chan S, Chand S *et al*. 2004. A set of BAC clones spanning the human genome. *Nucleic Acids Res* **32:** (12) 3651.
Li RG, Rimmer R, Buchwaldt L, Sharpe AG, Seguin-Swartz G, Coutu C, Hegedus DD. 2004. Interaction of *Sclerotinia sclerotiorum* with a resistant *Brassica napus* cultivar: Expressed sequence tag analysis identifies genes associated with fungal pathogenesis. *Fungal Genet Biol* **41:** (8) 735.
Oishi M, Gohma H, Lejukole HY, Taniguchi Y, Yamada T, Suzuki K, Shinkai H, Uenishi H, Yasue H, Sasaki Y. 2004. Generation of a total of 6483 expressed sequence tags from 60 day-old bovine whole fetus and fetal placenta. *Anim Biotechnol* **15:** (1) 1.
Tagu D, Prunier-Leterme N, Legeai F, Gauthier JP, Duclert A, Sabater-Munoz B, Bonhomme J, Simon JC. 2004. Annotated expressed sequence tags for studies of the regulation of reproductive modes in aphids. *Insect Biochem Mol Biol* **34:** (8) 809.
Varshney RK, Zhang HN, Potokina E, Stein N, Langridge P, Graner A. 2004. A simple hybridization-based strategy for the generation of non-redundant EST collections - A case study in barley (*Hordeum vulgare* L.). *Plant Sci* **167:** (3) 629.
Yao J, Burton JL, Saama P, Sipkovsky S, Coussens PM. 2003. Generation of EST and cDNA microarray resources for the study of bovine immunobiology. *Acta Vet Scand Suppl* **98:** 89.

### 9 Functional genomics

Colland F, Jacq X, Trouplin V, Mougin C, Groizeleau C, Hamburger A, Meil A, Wojcik J, Legrain P, Gauthier JM. 2004. Functional proteomics mapping of a human signaling pathway. *Genome Res* **14:** (7) 1324.
Cornell M, Paton NW, Oliver SG. 2004. A critical and integrated view of the yeast interactome. *Comp Funct Genom* **5:** (5) 382.
Dautry F, Ribet C. 2004. RNA interference: Towards a functional genomics in mammalian cells? (French). *MS Med Sci* **20:** (8-9) 815.
Gianinazzi-Pearson V, Brechenmacher L. 2004. Functional genomics of arbuscular mycorrhiza: Decoding the symbiotic cell programme. *Can J Bot* **82:** (8) 1228.
Granger L, Martin E, Segalat L. 2004. *Mos* as a tool for genome-wide insertional mutagenesis in *Caenorhabditis elegans*: Results of a pilot study - Online art. no. e117. *Nucleic Acids Res* **32:** (14) e117.
Jones DL, Petty J, Hoyle DC, Hayes A, Oliver SG, Riba-Garcia I, Gaskell SJ, Stateva L. 2004. Genome-wide analysis of the effects of heat shock on a *Saccharomyces cerevisiae* mutant with a constitutively activated cAMP-dependent pathway. *Comp Funct Genom* **5:** (5) 419.
Kang YS, Durfee T, Glasner JD, Qiu Y, Frisch D, Winterberg KM, Blattner F. 2004. Systematic mutagenesis of the *Escherichia coli* genome. *J Bacteriol* **186:** (15) 4921.
Korbel JO, Jensen LJ, Von Mering C, Bork P. 2004. Analysis of genomic context: Prediction of functional associations from conserved bidirectionally transcribed gene pairs. *Nat Biotechnol* **22:** (7) 911.
Kumar S, Fladung M. 2003. Forest tree transgenesis and functional genomics: From fast forward to reverse genetics. *Silvae Genetica* **52:** (5-6) 229.
Merritt J, Edwards JS. 2004. Assaying gene function by growth competition experiment. *Metab Eng* **6:** (3) 212.
Pickart MA, Sivasubbu S, Nielsen AL, Shriram S, King RA, Ekker SC. 2004. Functional genomics tools for the analysis of zebrafish pigment. *Pigment Cell Res* **17:** (5) 461.
Power PM, Jones RA, Beacham IR, Bucholtz C, Jennings MP. 2004. Whole genome analysis reveals a high incidence of non-optimal codons in secretory signal sequences of *Escherichia coli*. *Biochem Biophys Res Commun* **322:** (3) 1038.
Sato TK, Panda S, Miraglia LJ, Reyes TM, Rudic RD, McNamara P, Naik KA, Fitzgerald GA, Kay SA, Hogenesch JB. 2004. A functional genomics strategy reveals Rora as a component of the mammalian circadian clock. *Neuron* **43:** (4) 527.
Schattner P, Decatur WA, Davis CA, Ares M, Fournier MJ, Lowe TM. 2004. Genome-wide searching for pseudouridylation guide snoRNAs: Analysis of the *Saccharomyces cerevisiae* genome. *Nucleic Acids Res* **32:** (14) 4281.
Semizarov D, Kroeger P, Fesik S. 2004. siRNA-mediated gene silencing: A global genome view. *Nucleic Acids Res* **32:** (13) 3836.
Yang YZ, Peng H, Huang HM, Wu JX, Ha SR, Huang DF, Lu TG. 2004. Large-scale production of enhancer trapping lines for rice functional genomics. *Plant Sci* **167:** (2) 281.

### 10 Transcriptomics

Azumi K, Fujie M, Usami T, Miki Y, Satoh N. 2004. A cDNA microarray technique applied for analysis of global gene expression profiles in tributyltin-exposed ascidians. *Mar Environ Res* **58:** (2-5) 543.
Bartholomay LC, Cho WL, Rocheleau TA, Boyle JP, Beck ET, Fuchs JF, Liss P, Rusch M, Butler KM, Wu RCC *et al*. 2004. Description of the transcriptomes of immune response-activated hemocytes from the mosquito vectors *Aedes aegypti* and *Armigeres subalbatus*. *Infect Immun* **72:** (7) 4114.
Blum JL, Knoebl I, Larkin P, Kroll KJ, Denslow ND. 2004. Use of suppressive subtractive hybridization and cDNA arrays to discover patterns of altered gene expression in the liver of dihydrotestosterone and 11-ketotestosterone exposed adult male largemouth bass (*Micropterus salmoides*). *Mar Environ Res* **58:** (2-5) 565.
Bordin S, Amaral MEC, Anhe GF, Delghingaro-Augusto V, Cunha DA, Nicoletti-Carvalho JE, Boschero AC. 2004. Prolactin-modulated gene expression profiles in pancreatic islets from adult female rats. *Mol Cell Endocrinol* **220:** (1-2) 41.
Brown M, Davies IM, Moffat CF, Robinson C, Redshaw J, Craft JA. 2004. Identification of transcriptional effects of ethynyl oestradiol in male plaice (*Pleuronectes platessa*) by suppression subtractive hybridisation and a nylon macroarray. *Mar Environ Res* **58:** (2-5) 559.
Cavallaro S, D'Agata V, Alessi E, Coffa S, Alkon DL, Manickam P, Ciotti MT, Possenti R, Bonini P, Marlier L *et al*. 2004. Gene expression profiles of apoptotic neurons. *Genomics* **84:** (3) 485.
De Paepe A, Vuylsteke M, Van Hummelen P, Zabeau M, Van Der Straeten D. 2004. Transcriptional profiling by cDNA-AFLP and microarray analysis reveals novel insights into the early response to ethylene in *Arabidopsis*. *Plant J* **39:** (4) 537.
De Souza AA, Takita MA, Coletta HD, Caldana C, Yanai GM, Muto NH, De Oliveira RC, Nunes LR, Machado MA. 2004. Gene expression profile of the plant pathogen *Xylella fastidiosa* during biofilm formation *in vitro*. *FEMS Microbiol Lett* **237:** (2) 341.
Fazeli A, Affara NA, Hubank M, Holt WV. 2004. Sperm-induced modification of the oviductal gene expression profile after natural insemination in mice. *Biol Reprod* **71:** (1) 60.
Fujiwara S, Tanaka N, Kaneda T, Takayama S, Isogai A, Che FS. 2004. Rice cDNA microarray-based gene expression profiling of the response to flagellin perception in cultured rice cells. *Mol Plant Microbe Interact* **17:** (9) 986.
Getchell TV, Peng XJ, Green CP, Stromberg AJ, Chen KC, Mattson MP, Getchell ML. 2004. *In silico* analysis of gene expression profiles in the olfactory mucosae of aging senescence-accelerated mice. *J Neurosci Res* **77:** (3) 430.
Griffin MD, Xing NZ, Kumar R. 2004. Gene expression profiles in dendritic cells conditioned by 1$\alpha$,25-dihydroxyvitamin $D_3$ analog. *J Steroid Biochem Mol Biol* **89-90:** (1-5) 443.
Keen HL, Ryan MJ, Beyer A, Mathur S, Scheetz TE, Gackle BD, Faraci FM, Casavant TL, Sigmund CD. 2004. Gene expression profiling of

Copyright © 2005 John Wiley & Sons, Ltd. 

potential PPARγ target genes in mouse aorta. *Physiol Genomics* **18:** (1) 33.
Kinser S, Jia QS, Li MX, Laughter A, Cornwell PD, Corton JC, Pestka JJ. 2004. Gene expression profiling in spleens of deoxynivalenol-exposed mice: Immediate early genes as primary targets. *J Toxicol Environ Health A* **67:** (18) 1423.
Koskinen H, Pehkonen P, Vehniainen E, Krasnov A, Rexroad C, Afanasyev S, Molsa H, Oikari A. 2004. Response of rainbow trout transcriptome to model chemical contaminants. *Biochem Biophys Res Commun* **320:** (3) 745.
Lafarguette F, Leple JC, Dejardin A, Laurans F, Costa G, Lesage-Descauses MC, Pilate G. 2004. Poplar genes encoding fasciclin-like arabinogalactan proteins are highly expressed in tension wood. *New Phytol* **164:** (1) 107.
Lan LF, Chen W, Lai Y, Suo JF, Kong ZS, Li C, Lu Y, Zhang YJ, Zhao XY, Zhang XS *et al*. 2004. Monitoring of gene expression profiles and isolation of candidate genes involved in pollination and fertilization in rice (*Oryza sativa* L) with a 10K cDNA microarray. *Plant Mol Biol* **54:** (4) 471.
Lee H, Hur CG, Oh CJ, Kim HB, Park SY, An CS. 2004. Analysis of the root nodule-enhanced transcriptome in soybean. *Mol Cells* **18:** (1) 53.
Lefebvre C, Cocquerelle C, Vandenbulcke F, Hott D, Huot L, Lemoine Y, Salzet M. 2004. Transcriptomic analysis in the leech *Theromyzion tessulatum*: Involvement of cystatin B in innate immunity. *Biochem J* **380:** (3) 617.
Levanon EY, Eisenberg E, Yelin R, Nemzer S, Hallegger M, Shemesh R, Fligelman ZY, Shoshan A, Pollock SR, Sztybel D *et al*. 2004. Systematic identification of abundant A-to-I editing sites in the human transcriptome. *Nat Biotechnol* **22:** (8) 1001.
Li Y, Wasser S, Lim SG, Tan TMC. 2004. Genome-wide expression profiling of RNA interference of hepatitis B virus gene expression and replication. *Cell Mol Life Sci* **61:** (16) 2113.
Macaulay IC, Carr P, Farrugia R, Watkins NA. 2004. Analysing the platelet transcriptome. *Vox Sang* **87:** (Suppl 2) 42.
Manthey K, Krajinski F, Hohnjec N, Firnhaber C, Puhler A, Perlick AM, Kuster H. 2004. Transcriptome profiling in root nodules and arbuscular mycorrhiza identifies a collection of novel genes induced during *Medicago truncatula* root endosymbioses. *Mol Plant Microbe Interact* **17:** (10) 1063.
McDaniel LS, Thornton JA, McDaniel DO. 2004. Use of cDNA microarrays to analyze responses to pneumococcal virulence factors. *Indian J Med Res* **119:** (Suppl) 99.
McManus DP, Hu W, Brindley PJ, Feng Z, Han ZG. 2004. Schistosome transcriptome analysis at the cutting edge. *Trends Parasitol* **20:** (7) 301.
Meyers BC, Vu TH, Tej SS, Ghazal H, Matvienko M, Agrawal V, Ning JC, Haudenschild CD. 2004. Analysis of the transcriptional complexity of *Arabidopsis thaliana* by massively parallel signature sequencing. *Nat Biotechnol* **22:** (8) 1006.
Meyers BC, Tej SS, Vu TH, Haudenschild CD, Agrawal V, Edberg SB, Ghazal H, Decola S. 2004. The use of MPSS for whole-genome transcriptional analysis in *Arabidopsis*. *Genome Res* **14:** (8) 1641.
Morley M, Molony CM, Weber TM, Devlin JL, Ewens KG, Spielman RS, Cheung VG. 2004. Genetic analysis of genome-wide variation in human gene expression. *Nature* **430:** (7001) 743.
Nakagawa I, Nakata M, Kawabata S, Hamada S. 2004. Transcriptome analysis and gene expression profiles of early apoptosis-related genes in *Streptococcus pyogenes*-infected epithelial cells. *Cell Microbiol* **6:** (10) 939.
Nakagawa T, Schwartz JP. 2004. Gene expression profiles of reactive astrocytes in dopamine-depleted striatum. *Brain Pathol* **14:** (3) 275.
Nguyen TN, Ejaz AD, Brancieri MA, Mikula AM, Nelson KE, Gill SR, Noll KM. 2004. Whole-genome expression profiling of *Thermotoga maritima* in response to growth on sugars in a chemostat. *J Bacteriol* **186:** (14) 4824.
Ning W, Chu TJ, Li CJ, Choi AMK, Peters DG. 2004. Genome-wide analysis of the endothelial transcriptome under short-term chronic hypoxia. *Physiol Genomics* **18:** (1) 70.
Pappas CT, Sram J, Moskvin OV, Ivanov PS, Mackenzie RC, Choudhary M, Land ML, Larimer FW, Kaplan S, Gomelsky M. 2004. Construction and validation of the *Rhodobacter sphaeroides* 2.4.1 DNA microarray: Transcriptome flexibility at diverse growth modes. *J Bacteriol* **186:** (14) 4748.
Parani M, Rudrabhatla S, Myers R, Weirich H, Smith B, Leaman DW, Goldman SL. 2004. Microarray analysis of nitric oxide responsive transcripts in *Arabidopsis*. *Plant Biotechnol J* **2:** (4) 359.
Polen T, Wendisch VF. 2004. Genomewide expression analysis in amino acid-producing bacteria using DNA microarrays. *Appl Biochem Biotechnol* **118:** (1-3) 215.
Quiring R, Wittbrodt B, Henrich T, Ramialison M, Burgtorf C, Lehrach H, Wittbrodt J. 2004. Large-scale expression screening by automated whole-amount *in situ* hybridization. *Mech Dev* **121:** (7-8) 971.
Ranjan P, Kao YY, Jiang HY, Joshi CP, Harding SA, Tsai CJ. 2004. Suppression subtractive hybridization-mediated transcriptome analysis from multiple tissues of aspen (*Populus tremuloides*) altered in phenylpropanoid metabolism. *Planta* **219:** (4) 694.
Sato N, Ohmori M, Ikeuchi M, Tashiro K, Wolk CP, Kaneko T, Okada K, Tsuzuki M, Ehira S, Katoh H *et al*. 2004. Use of segment based microarray in the analysis of global gene expression in response to various environmental stresses in the cyanobacterium *Anabaena* sp PCC 7120. *J Gen Appl Microbiol Tokyo* **50:** (1) 1.
Schrader J, Nilsson J, Mellerowicz E, Berglund A, Nilsson P, Hertzberg M, Sandberg G. 2004. A high-resolution transcript profile across the wood-forming meristem of poplar identifies potential regulators of cambial stem cell identity. *Plant Cell* **16:** (9) 2278.
Smith CM, Rodriguez-Buey M, Karlsson J, Campbell MM. 2004. The response of the poplar transcriptome to wounding and subsequent infection by a viral pathogen. *New Phytol* **164:** (1) 123.
Suzuki T, Shin-I T, Kohara Y, Kasahara M. 2004. Transcriptome analysis of hagfish leukocytes: A framework for understanding the immune system of jawless fishes. *Dev Comp Immunol* **28:** (10) 993.
Tamaoki M, Matsuyama T, Nakajima N, Aono M, Kubo A, Saji H. 2004. A method for diagnosis of plant environmental stresses by gene expression profiling using a cDNA macroarray. *Environ Pollut* **131:** (1) 137.
Tao WJ, Mallard B, Karrow N, Bridle B. 2004. Construction and application of a bovine immune-endocrine cDNA microarray. *Vet Immunol Immunopathol* **101:** (1-2) 1.
Tomasinsig L, Scocchi M, Mettulio R, Zanetti M. 2004. Genome-wide transcriptional profiling of the *Escherichia coli* response to a proline-rich antimicrobial peptide. *Antimicrob Agents Chemother* **48:** (9) 3260.
Tong X, Campbell JW, Balazsi G, Kay KA, Wanner BL, Gerdes SY, Oltvai ZN. 2004. Genome-scale identification of conditionally essential genes in *E. coli* by DNA microarrays. *Biochem Biophys Res Commun* **322:** (1) 347.
Torres-Munoz JE, Van Waveren C, Keegan MG, Bookman RJ, Petito CK. 2004. Gene expression profiles in microdissected neurons from human hippocampal subregions. *Mol Brain Res* **127:** (1-2) 105.
Ventura M, Brussow H. 2004. Temporal transcription map of the virulent *Streptococcus thermophilus* bacteriophage Sfi19. *Appl Environ Microbiol* **70:** (8) 5041.
Verhagen BWM, Glazebrook J, Zhu T, Chang HS, Van Loon LC, Pieterse CMJ. 2004. The transcriptome of rhizobacteria-induced systemic resistance in *Arabidopsis*. *Mol Plant Microbe Interact* **17:** (8) 895.
Verjovski-Almeida S, Leite LCC, Dias-Neto E, Menck CFM, Wilson RA. 2004. Schistosome transcriptome: Insights and perspectives for functional genomics. *Trends Parasitol* **20:** (7) 304.
Wang QA, Brown S, Roos DS, Nussenzweig V, Bhanot P. 2004. Transcriptome of axenic liver stages of *Plasmodium yoelii*. *Mol Biochem Parasitol* **137:** (1) 161.
White JH. 2004. Profiling 1,25-dihydroxyvitamin $D_3$-regulated gene expression by microarray analysis. *J Steroid Biochem Mol Biol* **89-90:** (1-5) 239.
Yang SH, Perna NT, Cooksey DA, Okinaka Y, Lindow SE, Ibekwe AM, Keen NT, Yang CH. 2004. Genome-wide identification of plant-upregulated genes of *Erwinia chrysanthemi* 3937 using a GFP-based IVET leaf array. *Mol Plant Microbe Interact* **17:** (9) 999.
Yazaki J, Shimatani Z, Hashimoto A, Nagata Y, Fujii F, Kojima K, Suzuki K, Taya T, Tonouchi M, Nelson C, Kilkuchi S *et al*. 2004. Transcriptional profiling of genes responsive to abscisic acid and gibberellin in rice: Phenotyping and comparative analysis between rice and *Arabidopsis*. *Physiol Genomics* **17:** (2) 87.

### II Proteomics

Abbasi FM, Komatsu S. 2004. A proteomic approach to analyze salt-re-

Copyright © 2005 John Wiley & Sons, Ltd. 

sponsive proteins in rice leaf sheath. *Proteomics* **4:** (7) 2072.
Alfonso P, Rivera J, Hernaez B, Alonso C, Escribano JM. 2004. Identification of cellular proteins modified in response to African swine fever virus infection by proteomics. *Proteomics* **4:** (7) 2037.
Antelmann H, Sapolsky R, Miller B, Ferrari E, Chotani G, Weyler W, Gaertner A, Hecker M. 2004. Quantitative proteome profiling during the fermentation process of pleiotropic *Bacillus subtilis* mutants. *Proteomics* **4:** (8) 2408.
Arai M, Okumura K, Satake M, Shimizu T. 2004. Proteome-wide functional classification and identification of prokaryotic transmembrane proteins by transmembrane topology similarity comparison. *Protein Sci* **13:** (8) 2170.
Arnaud MC, Gazarian T, Rodriguez YP, Gazarian K, Sakanyan V. 2004. Array assessment of phage-displayed peptide mimics of human immunodeficiency virus type 1 gp41 immunodominant epitope: Binding to antibodies of infected individuals. *Proteomics* **4:** (7) 1959.
Avram D, Romijn EP, Pap EHW, Heck AJR, Wirtz KWA. 2004. Identification of proteins in activated human neutrophils susceptible to tyrosyl radical attack. A proteomic study using a tyrosylating fluorophore. *Proteomics* **4:** (8) 2397.
Baczek T. 2004. Fractionation of peptides and identification of proteins from *Saccharomyces cerevisiae* in proteomics with the use of reversed-phase capillary liquid chromatography and pI-based approach. *J Pharm Biomed Anal* **35:** (4) 895.
Bahrman N, Negroni L, Jaminon O, Le Gouis J. 2004. Wheat leaf proteome analysis using sequence data of proteins separated by two-dimensional electrophoresis. *Proteomics* **4:** (9) 2672.
Barzaghi D, Isbister JD, Lauer KP, Born TL. 2004. Use of surface-enhanced laser desorption/ionization-time of flight to explore bacterial proteomes. *Proteomics* **4:** (9) 2624.
Bestel-Corre G, Gianinazzi S, Dumas-Gaudot E. 2004. Impact of sewage studies on *Medicago truncatula* symbiotic proteome. *Phytochemistry* **65:** (11) 1651.
Blagoev B, Ong SE, Kratchmarova I, Mann M. 2004. Temporal analysis of phosphotyrosine-dependent signaling networks by quantitative proteomics. *Nat Biotechnol* **22:** (9) 1139.
Blonder J, Rodriguez-Galan MC, Chan KC, Lucas DA, Yu LR, Conrads TP, Issaq HJ, Young HA, Veenstra TD. 2004. Analysis of murine natural killer cell microsomal proteins using two-dimensional liquid chromatography coupled to tandem electrospray ionization mass spectrometry. *J Proteome Res* **3:** (4) 862.
Bouchal P, Precechtelova P, Zdrahal Z, Kucera I. 2004. Protein composition of *Paracoccus denitrificans* cells grown on various electron acceptors and in the presence of azide. *Proteomics* **4:** (9) 2662.
Boutell JM, Hart DJ, Godber BLJ, Kozlowski RZ, Blackburn JM. 2004. Functional protein microarrays for parallel characterisation of p53 mutants. *Proteomics* **4:** (7) 1950.
Brugiere S, Kowalski S, Ferro M, Seigneurin-Berny D, Miras S, Salvi D, Ravanel S, D'Herin P, Garin J, Bourguignon J *et al*. 2004. The hydrophobic proteome of mitochondrial membranes from *Arabidopsis* cell suspensions. *Phytochemistry* **65:** (12) 1693.
Campostrini N, Pascali J, Hadan B, Astner H, Marimpietri D, Pastorino F, Ponzoni M, Righetti PG. 2004. Proteomic analysis of an orthotopic neuroblastoma xenograft animal model. *J Chromatogr B* **808:** (2) 279.
Castillejo MA, Amiour N, Dumas-Gaudot E, Rubiales D, Jorrin JV. 2004. A proteomic approach to studying plant response to crenate broomrape (*Orobanche crenata*) in pea (*Pisum sativum*). *Phytochemistry* **65:** (12) 1817.
Chromy BA, Perkins J, Heidbrink JL, Gonzales AD, Murphy GA, Fitch JP, McCutchen-Maloney SL. 2004. Proteomic characterization of host response to *Yersinia pestis* and near neighbors. *Biochem Biophys Res Commun* **320:** (2) 474.
Coaker GL, Willard B, Kinter M, Stockinger EJ, Francis DM. 2004. Proteomic analysis of resistance mediated by Rcm 2.0 and Rcm 5.1, two loci controlling resistance to bacterial canker of tomato. *Mol Plant Microbe Interact* **17:** (9) 1019.
Doherty MK, McLean L, Hayter JR, Pratt JM, Robertson DHL, El-Shafei A, Gaskell SJ, Beynon RJ. 2004. The proteome of chicken skeletal muscle: Changes in soluble protein expression during growth in a layer strain. *Proteomics* **4:** (7) 2082.
Dumur J, Jahier J, Bancel E, Lauriere M, Bernard M, Branlard G. 2004. Proteomic analysis of aneuploid lines in the homeologous group 1 of the hexaploid wheat cultivar Courtot. *Proteomics* **4:** (9) 2685.
Dunkley TPJ, Dupree P, Watson RB, Lilley KS. 2004. The use of isotope-coded affinity tags (ICAT) to study organelle proteomes in *Arabidopsis thaliana*. *Biochem Soc Trans* **32:** (3) 520.
Finnie C, Steenholdt T, Noguera OR, Knudsen S, Larsen J, Brinch-Pedersen H, Holm PB, Olsen O, Svensson B. 2004. Environmental and transgene expression effects on the barley seed proteome. *Phytochemistry* **65:** (11) 1619.
Fuentes M, Segura RL, Abian O, Betancor L, Hidalgo A, Mateo C, Fernandez-Lafuente R, Guisan JM. 2004. Determination of protein-protein interactions through aldehyde-dextran intermolecular cross-linking. *Proteomics* **4:** (9) 2602.
Fujii K, Nakano T, Kawamura T, Usui F, Bando Y, Wang R, Nishimura T. 2004. Multidimensional protein profiling technology and its application to human plasma proteome. *J Proteome Res* **3:** (4) 712.
Ge Y, Molloy MP, Chamberlain JS, Andrews PC. 2004. Differential expression of the skeletal muscle proteome in *mdx* mice at different ages. *Electrophoresis* **25:** (15) 2576.
Gelfi C, Vigano A, Ripamonti M, Wait R, Begum S, Biguzzi E, Castaman G, Faioni EM. 2004. A proteomic analysis of changes in prothrombin and plasma proteins associated with the G20210A mutation. *Proteomics* **4:** (7) 2151.
Goodchild A, Saunders NFW, Ertan H, Raftery M, Guilhaus M, Curmi PMG, Cavicchioli R. 2004. A proteomic determination of cold adaptation in the Antarctic archaeon, *Methanococcoides burtonii*. *Mol Microbiol* **53:** (1) 309.
Greetham D, Morgan C, Campbell AM, Van Rossum AJ, Barrett J, Brophy PM. 2004. Evidence of glutathione transferase complexing and signaling in the model nematode *Caenorhabditis elegans* using a pull-down proteomic assay. *Proteomics* **4:** (7) 1989.
Han J, Schey KL. 2004. Proteolysis and mass spectrometric analysis of an integral membrane: Aquaporin O. *J Proteome Res* **3:** (4) 807.
Hayduk EJ, Choe LH, Lee KH. 2004. A two-dimensional electrophoresis map of Chinese hamster ovary cell proteins based on fluorescence staining. *Electrophoresis* **25:** (15) 2545.
Hedegaard J, Horn P, Lametsch R, Moller HS, Roepstorff P, Bendixen C, Bendixen E. 2004. UDP-glucose pyrophosphorylase is upregulated in carriers of the porcine *RN*⁻ mutation in the AMP-activated protein kinase. *Proteomics* **4:** (8) 2448.
Heinemeyer J, Eubel H, Wehmhoner D, Jansch L, Braun HP. 2004. Proteomic approach to characterize the supramolecular organization of photosystems in higher plants. *Phytochemistry* **65:** (12) 1683.
Hogarth CJ, Fitzpatrick JL, Nolan AM, Young FJ, Pitt A, Eckersall PD. 2004. Differential protein composition of bovine whey: A comparison of whey from healthy animals and from those with clinical mastitis. *Proteomics* **4:** (7) 2094.
Hozumi A, Satouh Y, Ishibe D, Kaizu M, Konno A, Ushimaru Y, Toda T, Inaba K. 2004. Local database and the search program for proteomic analysis of sperm proteins in the ascidian *Ciona intestinalis*. *Biochem Biophys Res Commun* **319:** (4) 1241.
Huang CM, Foster KW, DeSilva TS, Van Kampen KR, Elmets CA, Tang DCC. 2004. Identification of *Bacillus anthracis* proteins associated with germination and early outgrowth by proteomic profiling of anthrax spores. *Proteomics* **4:** (9) 2653.
Imin N, De Jong F, Mathesius U, Van Noorden G, Saeed NA, Wang XD, Rose RJ, Rolfe BG. 2004. Proteome reference maps of *Medicago truncatula* embryogenic cell cultures generated from single protoplasts. *Proteomics* **4:** (7) 1883.
Islam MK, Miyoshi T, Yokomizo Y, Tsuji N. 2004. The proteome expression patterns in adult *Ascaris suum* under exposure to aerobic/anaerobic environments analyzed by two-dimensional electrophoresis. *Parasitol Res* **93:** (2) 96.
Jones AME, Thomas V, Truman B, Lilley K, Mansfield J, Grant M. 2004. Specific changes in the *Arabidopsis* proteome in response to bacterial challenge: Differentiating basal and *R*-gene mediated resistance. *Phytochemistry* **65:** (12) 1805.
Kim YH, Han KY, Lee K, Heo JH, Kang HA, Lee J. 2004. Comparative proteome analysis of *Hansenula polymorpha* DL1 and A16. *Proteomics* **4:** (7) 2005.
Kim YH, Park JS, Cho JY, Cho KM, Park YH, Lee J. 2004. Proteomic response analysis of a threonine-overproducing mutant of *Escherichia coli*. *Biochem J* **381:** (3) 823.
Kramer A, Feilner T, Possling A, Radchuk V, Weschke W, Burkle L, Kersten B. 2004. Identification of barley CK2$\alpha$ targets by using the protein microarray technology. *Phytochemistry* **65:** (12) 1777.
Kristensen BK, Askerlund P, Bykova NV, Egsgaard H, Moller IM. 2004.

Copyright © 2005 John Wiley & Sons, Ltd. 

Identification of oxidised proteins in the matrix of rice leaf mitochondria by immunoprecipitation and two-dimensional liquid chromatography-tandem mass spectrometry. *Phytochemistry* **65:** (12) 1839.
Kuznetsov VY, Ivanov YD, Archakov AI. 2004. Atomic force microscopy revelation of molecular complexes in the multiprotein cytochrome P4502B4-containing system. *Proteomics* **4:** (8) 2390.
Le Bihan MC, Tarelli E, Coulton GR. 2004. Evaluation of an integrated strategy for proteomic profiling of skeletal muscle. *Proteomics* **4:** (9) 2739.
Lee HW, Choe YH, Kim DK, Jung SY, Lee NG. 2004. Proteomic analysis of a ferric uptake regulator mutant of *Helicobacter pylori*: Regulation of *Helicobacter pylori* gene expression by ferric uptake regulator and iron. *Proteomics* **4:** (7) 2014.
Liska AJ. 2004. The morality of problem selection in proteomics. *Proteomics* **4:** (7) 1929.
Liska AJ, Popov AV, Sunyaev S, Coughlin P, Habermann B, Shevchenko A, Bork P, Karsenti E, Shevchenko A. 2004. Homology-based functional proteomics by mass spectrometry: Application to the *Xenopus* microtubule-associated proteome. *Proteomics* **4:** (9) 2707.
Loschelder H, Homann A, Ogrzewalla K, Link G. 2004. Proteomics-based sequence analysis of plant gene expression - The chloroplast transcription apparatus. *Phytochemistry* **65:** (12) 1785.
Marchand C, Le Marechal P, Meyer Y, Miginiac-Maslow M, Issakidis-Bourguet E, Decottignies P. 2004. New targets of *Arabidopsis* thioredoxins revealed by proteomic analysis. *Proteomics* **4:** (9) 2696.
Mechin V, Balliau T, Chateau-Joubert S, Davanture M, Langella O, Negroni L, Prioul JL, Thevenot C, Zivy M, Damerval C. 2004. A two-dimensional proteome map of maize endosperm. *Phytochemistry* **65:** (11) 1609.
Meek SEM, Lane WS, Piwnica-Worms H. 2004. Comprehensive proteomic analysis of interphase and mitotic 14-3-3-binding proteins. *J Biol Chem* **279:** (31) 32046.
Millar AH. 2004. Location, location, location: Surveying the intracellular real estate through proteomics in plants. *Funct Plant Biol* **31:** (6) 563.
Millar AH, Trend AE, Heazlewood JL. 2004. Changes in the mitochondrial proteome during the anoxia to air transition in rice focus around cytochrome-containing respiratory complexes. *J Biol Chem* **279:** (38) 39471.
Miranda G, Mahe MF, Leroux C, Martin P. 2004. Proteomic tools to characterize the protein fraction of Equidae milk. *Proteomics* **4:** (8) 2496.
Mohanty BP, Mohanty S. 2004. Proteomics and human proteome organization. *Natl Acad Sci Lett* **27:** (7-8) 249.
Moini M, Huang HL. 2004. Application of capillary electrophoresis/electrospray ionization-mass spectrometry to subcellular proteomics of *Escherichia coli* ribosomal proteins. *Electrophoresis* **25:** (13) 1981.
Mooney BP, Thelen JJ. 2004. High-throughput peptide mass fingerprinting of soybean seed proteins: Automated workflow and utility of UniGene expressed sequence tag databases for protein identification. *Phytochemistry* **65:** (12) 1733.
Nelson RW, Nedelkov D, Tubbs KA, Kiernan UA. 2004. Quantitative mass spectrometric immunoassay of insulin like growth factor 1. *J Proteome Res* **3:** (4) 851.
Nuhse TS, Stensballe A, Jensen ON, Peck SC. 2004. Phosphoproteomics of the *Arabidopsis* plasma membrane and a new phosphorylation site database. *Plant Cell* **16:** (9) 2394.
Peng XX, Ye XT, Wang SY. 2004. Identification of novel immunogenic proteins of *Shigella flexneri* 2a by proteomic methodologies. *Vaccine* **22:** (21-22) 2750.
Poon HF, Castegna A, Farr SA, Thongboonkerd V, Lynn BC, Banks WA, Morley JE, Klein JB, Butterfield DA. 2004. Quantitative proteomics analysis of specific protein expression and oxidative modification in aged senescence-accelerated-prone 8 mice brain. *Neuroscience* **126:** (4) 915.
Rao PSS, Yamada Y, Tan YP, Leung KY. 2004. Use of proteomics to identify novel virulence determinants that are required for *Edwardsiella tarda* pathogenesis. *Mol Microbiol* **53:** (2) 573.
Rao RS, Visuri SR, McBride MT, Albala JS, Matthews DL, Coleman MA. 2004. Comparison of multiplexed techniques for detection of bacterial and viral proteins. *J Proteome Res* **3:** (4) 736.
Roncada P, Carta F, Zannotti M, Malagutti L, Sciaraffia F, Greppi GF. 2004. Swine ochratoxicosis: Proteomic investigation of epatic bioindicators. *Vet Res Commun* **28:** (1 Suppl 1) 371.
Saitoh N, Spahr CS, Patterson SD, Bubulya P, Neuwald AF, Spector DL. 2004. Proteomic analysis of interchromatin granule clusters. *Mol Biol Cell* **15:** (8) 3876.
Santos PM, Benndorf D, Sa-Correia I. 2004. Insights into *Pseudomonas putida* KT2440 response to phenol-induced stress by quantitative proteomics. *Proteomics* **4:** (9) 2640.
Sarnighausen E, Wurtz V, Heintz D, Van Dorsselaer A, Reski R. 2004. Mapping of the *Physcomitrella patens* proteome. *Phytochemistry* **65:** (11) 1589.
Sawicki G, Jugdutt BI. 2004. Detection of regional changes in protein levels in the *in vivo* canine model of acute heart failure following ischemia-reperfusion injury: Functional proteomics studies. *Proteomics* **4:** (7) 2195.
Sazuka T, Keta S, Shiratake K, Yamaki S, Shibata D. 2004. A proteomic approach to identification of transmembrane proteins and membrane-anchored proteins of *Arabidopsis thaliana* by peptide sequencing. *DNA Res* **11:** (2) 101.
Schlesier B, Berna A, Bernier F, Mock HP. 2004. Proteome analysis differentiates between two highly homologues germin-like proteins in *Arabidopsis thaliana* ecotypes Col-0 and Ws-2. *Phytochemistry* **65:** (11) 1565.
Schmid GM, Converset V, Walter N, Sennitt MV, Leung KY, Byers H, Ward M, Hochstrasser DF, Cawthorne MA, Sanchez JC. 2004. Effect of high-fat diet on the expression of proteins in muscle, adipose tissues, and liver of C57BL/6 mice. *Proteomics* **4:** (8) 2270.
Schrattenholz A, Klemm M, Cahill M. 2004. Potential of comprehensive toxico-proteomics: Quantitative and differential mining of functional proteomes from native samples. *ATLA* **32:** (Suppl 1A) 123.
Secko DM, Insall RH, Spiegelman GB, Weeks G. 2004. The identification of *Dictyostelium* phosphoproteins altered in response to the activation of RasG. *Proteomics* **4:** (9) 2629.
Shimaoka T, Ohnishi M, Sazuka T, Mitsuhashi N, Hara-Nishimura I, Shimazaki KI, Maeshima M, Yokota A, Tomizawa KI, Mimura T. 2004. Isolation of intact vacuoles and proteomic analysis of tonoplast from suspension-cultured cells of *Arabidopsis thaliana*. *Plant Cell Physiol* **45:** (6) 672.
Skop AR, Liu HB, Yates J, Meyer BJ, Heald R. 2004. Dissection of the mammalian midbody proteome reveals conserved cytokinesis mechanisms. *Science* **305:** (5680) 61.
Slabas AR, Ndimba B, Simon WJ, Chivasa S. 2004. Proteomic analysis of the *Arabidopsis* cell wall reveals unexpected proteins with new cellular locations. *Biochem Soc Trans* **32:** (3) 524.
Sule A, Vanrobaeys F, Hajos G, Van Beeumen J, Devreese B. 2004. Proteomic analysis of small heat shock protein isoforms in barley shoots. *Phytochemistry* **65:** (12) 1853.
Swidzinski JA, Leaver CJ, Sweetlove LJ. 2004. A proteomic analysis of plant programmed cell death. *Phytochemistry* **65:** (12) 1829.
Tanaka N, Fujita M, Handa H, Murayama S, Uemura M, Kawamura Y, Mitsui T, Mikami S, Tozawa Y, Yoshinaga T *et al*. 2004. Proteomics of the rice cell: Systematic identification of the protein populations in subcellular compartments. *Mol Genet Genomics* **271:** (5) 566.
Tenzer A, Hofstetter B, Sauser C, Bodis S, Schubiger AP, Bonny C, Pruschy M. 2004. Profiling treatment-specific post-translational modifications in a complex proteome with subtractive substrate phage display. *Proteomics* **4:** (9) 2796.
Trisiriroj A, Jeyachok N, Chen ST. 2004. Proteomics characterization of different bran proteins between aromatic and nonaromatic rice (*Oryza sativa* L. ssp *indica*). *Proteomics* **4:** (7) 2047.
Tseng CF, Lin CC, Huang HY, Liu HC, Mao SJT. 2004. Antioxidant role of human haptoglobin. *Proteomics* **4:** (8) 2221.
Valot B, Gianinazzi S, Eliane DG. 2004. Sub-cellular proteomic analysis of a *Medicago truncatula* root microsomal fraction. *Phytochemistry* **65:** (12) 1721.
Vertegaal ACO, Ogg SC, Jaffray E, Rodriguez MS, Hay RT, Andersen JS, Mann M, Lamond AI. 2004. A proteomic study of SUMO-2 target proteins. *J Biol Chem* **279:** (32) 33791.
Vilain S, Cosette P, Hubert M, Lange C, Junter GA, Jouenne T. 2004. Proteomic analysis of agar gel-entrapped *Pseudomonas aeruginosa*. *Proteomics* **4:** (7) 1996.
Walz C, Giavalisco P, Schad M, Juenger M, Klose J, Kehr J. 2004. Proteomics of curcurbit phloem exudate reveals a network of defence proteins. *Phytochemistry* **65:** (12) 1795.
Wang YQ, Zhang PB, Fujii H, Banno Y, Yamamoto K, Aso Y. 2004. Proteomic studies of lipopolysaccharide-induced polypeptides in the silkworm, *Bombyx mori*. *Biosci Biotechnol Biochem* **68:** (8) 1821.

Copyright © 2005 John Wiley & Sons, Ltd. 

Watson BS, Lei ZT, Dixon RA, Sumner LW. 2004. Proteomics of *Medicago sativa* cell walls. *Phytochemistry* **65:** (12) 1709.
Whitelegge JP, Katz JE, Pihakari KA, Hale R, Aguilera R, Gomez SM, Faull KF, Vavilin D, Vermaas W. 2004. Subtle modification of isotope ratio proteomics: An integrated strategy for expression proteomics. *Phytochemistry* **65:** (11) 1507.
Wilmarth PA, Taube JR, Riviere MA, Duncan MK, David LL. 2004. Proteomic and sequence analysis of chicken lens crystallins reveals alternate splicing and translational forms of βB2 and βA2 crystallins. *Invest Ophthalmol Vis Sci* **45:** (8) 2705.
Wong JH, Cal N, Balmer Y, Tanaka CK, Vensel WH, Hurkman WJ, Buchanan BB. 2004. Thioredoxin targets of developing wheat seeds identified by complementary proteomic approaches. *Phytochemistry* **65:** (11) 1629.
Yajima W, Hall JC, Kav NNV. 2004. Proteome-level differences between auxinic-herbicide-susceptible and -resistant wild mustard (*Sinapis arvensis* L). *J Agric Food Chem* **52:** (16) 5063.
Yin ZK, Stead D, Selway L, Walker J, Riba-Garcia I, McInerney T, Gaskell S, Oliver SG, Cash P, Brown AJP. 2004. Proteomic response to amino acid starvation in *Candida albicans* and *Saccharomyces cerevisiae*. *Proteomics* **4:** (8) 2425.
Yuan Q, Fontenele-Neto JD, Fricker LD. 2004. Effect of voluntary exercise on genetically obese $Cpe^{fat/fat}$ mice: Quantitative proteomics of serum. *Obes Res* **12:** (7) 1179.
Yun HS, Jeong W, Do SH, Jeong DH, Jung YR, Park JK, Cho EM, Jeong KS. 2004. Proteome analysis by bio-active ceramic water in rat liver: Contribution to antioxidant enzyme expression, SOD I. *Biochem Biophys Res Commun* **320:** (3) 852.
Zhang CJ, Doherty-Kirby A, Van Huystee R, Lajoie G. 2004. Investigation of cationic peanut peroxidase glycans by electrospray ionization mass spectrometry. *Phytochemistry* **65:** (11) 1575.
Zhang O, Riechers DE. 2004. Proteomic characterization of herbicide safener-induced proteins in the coleoptile of *Triticum tauschii* seedlings. *Proteomics* **4:** (7) 2058.
Zhao B, Yeo CC, Lee CC, Geng A, Chew FT, Poh CL. 2004. Proteome analysis of gentisate-induced response in *Pseudomonas alcaligenes* NCIB 9867. *Proteomics* **4:** (7) 2028.
Zhou Y, Gu GY, Goodlett DR, Zhang T, Pan C, Montine TJ, Montine KS, Aebersold RH, Zhang J. 2004. Analysis of α-synuclein-associated proteins by quantitative proteomics. *J Biol Chem* **279:** (37) 39155.
Zolodz MD, Wood KV, Regnier FE, Geahlen RL. 2004. New approach for analysis of the phosphotyrosine proteome and its application to the chicken B cell line, DT40. *J Proteome Res* **3:** (4) 743.

### I2 Protein structural genomics

Chou KC. 2004. Insights from modeling three-dimensional structures of the human potassium and sodium channels. *J Proteome Res* **3:** (4) 856.
Cornilescu G, Cornilescu CC, Zhao Q, Frederick RO, Peterson FC, Thao S, Markley JL. 2004. Solution structure of a homodimeric hypothetical protein, At5g22580, a structural genomics target from *Arabidopsis thaliana*. *J Biomol NMR* **29:** (3) 387.
Liu JF, Hegyi H, Acton TB, Montelione GT, Rost B. 2004. Automatic target selection for structural genomics on eukaryotes. *Proteins* **56:** (2) 188.
O'Toole N, Grabowski M, Otwinowski Z, Minor W, Cygler M. 2004. The structural genomics experimental pipeline: Insights from global target lists. *Proteins* **56:** (2) 201.
Stark A, Shkumatov A, Russell RB. 2004. Finding functional sites in structural genomics proteins. *Structure* **12:** (8) 1405.

### I3 Metabolomics

Bro C, Nielsen J. 2004. Impact of 'ome' analyses on inverse metabolic engineering. *Metab Eng* **6:** (3) 204.
Denef VJ, Park J, Tsoi TV, Rouillard JM, Zhang H, Wibbenmeyer JA, Verstraete W, Gulari E, Hashsham SA, Tiedje JM. 2004. Biphenyl and benzoate metabolism in a genomic context: Outlining genome-wide metabolic networks in *Burkholderia xenovorans* LB400. *Appl Environ Microbiol* **70:** (8) 4961.
Djordjevic MA. 2004. *Sinorhizobium meliloti* metabolism in the root nodule: A proteomic perspective. *Proteomics* **4:** (7) 1859.
Duarte NC, Herrgard MJ, Palsson BO. 2004. Reconstruction and validation of *Saccharomyces cerevisiae* iND750, a fully compartmentalized genome-scale metabolic model. *Genome Res* **14:** (7) 1298.
Etchebarne BE, Nobis W, Allen MS, Van de Haar MJ. 2004. Design of a bovine metabolism oligonucleotide gene array. *J Anim Feed Sci* **13:** (Suppl 1) 385.
Griffin JL, Bonney SA, Mann C, Hebbachi AM, Gibbons GF, Nicholson JK, Shoulders CC, Scott J. 2004. An integrated reverse functional genomic and metabolic approach to understanding orotic acid-induced fatty liver. *Physiol Genomics* **17:** (2) 140.
Gullberg J, Jonsson P, Nordstrom A, Sjostrom M, Moritz T. 2004. Design of experiments: An efficient strategy to identify factors influencing extraction and derivatization of *Arabidopsis thaliana* samples in metabolomic studies with gas chromatography/mass spectrometry. *Anal Biochem* **331:** (2) 283.
Hirai MY, Yano M, Goodenowe DB, Kanaya S, Kimura T, Awazuhara M, Arita M, Fujiwara T, Saito K. 2004. Integration of transcriptomics and metabolomics for understanding of global responses to nutritional stresses in *Arabidopsis thaliana*. *Proc Natl Acad Sci U S A* **101:** (27) 10205.
Kabir MM, Shimizu K. 2004. Metabolic regulation analysis of *icd*-gene knockout *Escherichia coli* based on 2D electrophoresis with MALDI-TOF mass spectrometry and enzyme activity measurements. *Appl Microbiol Biotechnol* **65:** (1) 84.
Li Z, Chan C. 2004. Integrating gene expression and metabolic profiles. *J Biol Chem* **279:** (26) 27124.
Maeda H, Sano M, Maruyama Y, Tanno T, Akao T, Totsuka Y, Endo M, Sakurada R, Yamagata Y, Machida M *et al*. 2004. Transcriptional analysis of genes for energy catabolism and hydrolytic enzymes in the filamentous fungus *Aspergillus oryzae* using cDNA microarrays and expressed sequence tags. *Appl Microbiol Biotechnol* **65:** (1) 74.
Price J, Laxmi A, St Martin SK, Jang JC. 2004. Global transcription profiling reveals multiple sugar signal transduction mechanisms in *Arabidopsis*. *Plant Cell* **16:** (8) 2128.
Sawasaki T, Hasegawa Y, Morishita R, Seki M, Shinozaki K, Endo Y. 2004. Genome-scale, biochemical annotation method based on the wheat germ cell-free protein synthesis system. *Phytochemistry* **65:** (11) 1549.
Vo TD, Greenberg HJ, Palsson BO. 2004. Reconstruction and functional characterization of the human mitochondrial metabolic network based on proteomic and biochemical data. *J Biol Chem* **279:** (38) 39532.
Wienkoop S, Zoeller D, Ebert B, Simon-Rosin U, Fisahn J, Glinski M, Weckwerth W. 2004. Cell-specific protein profiling in *Arabidopsis thaliana* trichomes: Identification of trichome-located proteins involved in sulfur metabolism and detoxification. *Phytochemistry* **65:** (11) 1641.

### I4 Genomic approaches to development

Amsterdam A, Nissen RM, Sun ZX, Swindell EC, Farrington S, Hopkins N. 2004. Identification of 315 genes essential for early zebrafish development. *Proc Natl Acad Sci U S A* **101:** (35) 12792.
Bantscheff M, Ringel B, Madi A, Schnabel R, Glocker MO, Thiesen HJ. 2004. Differential proteome analysis and mass spectrometric characterization of germ line development-related proteins of *Caenorhabditis elegans*. *Proteomics* **4:** (8) 2283.
Bourne S, Polak JM, Hughes SPF, Buttery LDK. 2004. Osteogenic differentiation of mouse embryonic stem cells: Differential gene expression analysis by cDNA microarray and purification of osteoblasts by cadherin-11 magnetically activated cell sorting. *Tissue Eng* **10:** (5-6) 796.
Casu RE, Dimmock CM, Chapman SC, Grof CPL, McIntyre CL, Bonnett GD, Manners JM. 2004. Identification of differentially expressed transcripts from maturing stem of sugarcane by *in silico* analysis of stem expressed sequence tags and gene expression profiling. *Plant Mol Biol* **54:** (4) 503.
Chen HW, Yu SL, Chen WJ, Yang PC, Chien CT, Chou HY, Li HN, Peck K, Huang CH, Lin FY *et al*. 2004. Dynamic changes of gene expression profiles during postnatal development of the heart in mice. *Heart* **90:** (8) 927.
Dobson AT, Raja R, Abeyta MJ, Taylor T, Shen S, Haqq C, Pera RAR. 2004. The unique transcriptome through day 3 of human preimplantation development. *Hum Mol Genet* **13:** (14) 1461.
Finnie C, Maeda K, Ostergaard O, Bak-Jensen KS, Larsen J, Svensson B. 2004. Aspects of the barley seed proteome during development and

Copyright © 2005 John Wiley & Sons, Ltd. 

germination. *Biochem Soc Trans* **32:** (3) 517.
Fonseca S, Hackler L, Zvara A, Ferreira S, Balde A, Dudits D, Pals MS, Puskas LG. 2004. Monitoring gene expression along pear fruit development, ripening and senescence using cDNA microarrays. *Plant Sci* **167:** (3) 457.
Furutani-Seiki M, Sasadoa T, Morinaga C, Suwa H, Niwa K, Yoda H, Deguchi T, Hirose Y, Yasuoka A, Henrich T *et al*. 2004. A systematic genome-wide screen for mutations affecting organogenesis in medaka, *Oryzias latipes*. *Mech Dev* **121:** (7-8) 647.
Hamatani T, Carter MG, Sharov AA, Ko MSH. 2004. Dynamics of global gene expression changes during mouse preimplantation development. *Dev Cell* **6:** (1) 117.
Imin N, Kerim T, Rolfe BG, Weinman JJ. 2004. Effect of early cold stress on the maturation of rice anthers. *Proteomics* **4:** (7) 1873.
Jorge EC, Monteiro-Vitorello CB, Alves HJ, Silva CS, Balan RG, Patricio M, Coutinho LL. 2004. EST analysis of mRNAs expressed during embryogenesis in *Gallus gallus*. *Int J Dev Biol* **48:** (4) 333.
Katogi R, Nakatani Y, Shin-i T, Kohara Y, Inohaya K, Kudo A. 2004. Large-scale analysis of the genes involved in fin regeneration and blastema formation in the medaka, *Oryzias latipes*. *Mech Dev* **121:** (7-8) 861.
Kimura T, Jindo T, Narita T, Naruse K, Kobayashi D, Shin-I T, Kitagawa T, Sakaguchi T, Mitani H, Shima A *et al*. 2004. Large-scale isolation of ESTs from medaka embryos and its application to medaka developmental genetics. *Mech Dev* **121:** (7-8) 915.
Li Y, Chia JM, Bartfai R, Christoffels A, Yue GH, Ding K, Ho MY, Hill JA, Stupka E, Orban L. 2004. Comparative analysis of the testis and ovary transcriptomes in zebrafish by combining experimental and computational tools. *Comp Funct Genom* **5:** (5) 403.
Liu JY, Blaylock LA, Harrison MJ. 2004. cDNA arrays as a tool to identify mycorrhiza-regulated genes: Identification of mycorrhiza-induced genes that encode or generate signaling molecules implicated in the control of root growth. *Can J Bot* **82:** (8) 1177.
Lu J, Qian J, Izvolsky KI, Cardoso WV. 2004. Global analysis of genes differentially expressed in branching and non-branching regions of the mouse embryonic lung. *Dev Biol* **273:** (2) 418.
Lurin C, Andres C, Aubourg S, Bellaoui M, Bitton F, Bruyere C, Caboche M, Debast C, Gualberto J, Hoffmann B *et al*. 2004. Genome-wide analysis of *Arabidopsis* pentatricopeptide repeat proteins reveals their essential role in organelle biogenesis. *Plant Cell* **16:** (8) 2089.
Matyash A, Chung HR, Jackle H. 2004. Genome-wide mapping of *in vivo* targets of the *Drosophila* transcription factor Kruppel. *J Biol Chem* **279:** (29) 30689.
Ote M, Mita K, Kawasaki H, Seki M, Nohata J, Kobayashi M, Shimada T. 2004. Microarray analysis of gene expression profiles in wing discs of *Bombyx mori* during pupal ecdysis. *Insect Biochem Mol Biol* **34:** (8) 775.
Print C, Valtola R, Lessan K, Malik S, Smith S. 2004. Soluble factors from human endometrium promote angiogenesis and regulate the endothelial cell transcriptome. *Hum Reprod* **19:** (10) 2356.
Shim MH, Hoover A, Blake N, Drachman JG, Reems JA. 2004. Gene expression profile of primary human $CD34^{+}CD38^{lo}$cells differentiating along the megakaryocyte lineage. *Exp Hematol* **32:** (7) 638.
Shima JE, McLean DJ, McCarrey JR, Griswold MD. 2004. The murine testicular transcriptome: Characterizing gene expression in the testis during the progression of spermatogenesis. *Biol Reprod* **71:** (1) 319.
Taji T, Seki M, Satou M, Sakurai T, Kobayashi M, Ishiyama K, Narusaka Y, Narusaka M, Zhu JK, Shinozaki K. 2004. Transcriptional programs of early reproductive stages in *Arabidopsis*. *Plant Physiol* **135:** (3) 1765.
Tzafrir I, Pena-Muralla R, Dickerman A, Berg M, Rogers R, Hutchens S, Sweeney TC, McElver J, Aux G, Patton D *et al*. 2004. Identification of genes required for embryo development in *Arabidopsis*. *Plant Physiol* **135:** (3) 1206.
Wang QT, Piotrowska K, Ciemerych MA, Milenkovic L, Scott MP, Davis RW, Zernicka-Goetz M. 2004. A genome-wide study of gene activity reveals developmental signaling pathways in the preimplantation mouse embryo. *Dev Cell* **6:** (1) 133.
Yamaguchi T, Nakayama K, Hayashi T, Yazaki J, Kishimoto N, Kikuchi S, Koike S. 2004. cDNA microarray analysis of rice anther genes under chilling stress at the microsporogenesis stage revealed two genes with DNA transposon *Castaway* in the 5′-flanking region. *Biosci Biotechnol Biochem* **68:** (6) 1315.
Zeng FY, Baldwin DA, Schultz RM. 2004. Transcript profiling during preimplantation mouse development. *Dev Biol* **272:** (2) 483.

### I5 Technological advances

Allet N, Barrillat N, Baussant T, Boiteau C, Botti P, Bougueleret L, Budin N, Canet D, Carraud S, Chiappe D *et al*. 2004. *In vitro* and *in silico* processes to identify differentially expressed proteins. *Proteomics* **4:** (8) 2333.
Boja ES, Sokoloski EA, Fales HM. 2004. Divinyl sulfone as a postdigestion modifier for enhancing the $a_1$ ion in MS/MS and postsource decay: Potential applications in proteomics. *Anal Chem* **76:** (14) 3958.
Bredemeyer AJ, Lewis RM, Malone JP, Davis AE, Gross J, Townsend RR, Ley TJ. 2004. A proteomic approach for the discovery of protease substrates. *Proc Natl Acad Sci U S A* **101:** (32) 11785.
Cha T, Guo A, Jun Y, Pei DQ, Zhu XY. 2004. Immobilization of oriented protein molecules on poly(ethylene glycol)-coated Si(111). *Proteomics* **4:** (7) 1965.
Chernokalskaya E, Gutierrez S, Pitt AM, Leonard JT. 2004. Ultrafiltration for proteomic sample preparation. *Electrophoresis* **25:** (15) 2461.
Chevalier F, Rofidal V, Vanova P, Bergoin A, Rossignol M. 2004. Proteomic capacity of recent fluorescent dyes for protein staining. *Phytochemistry* **65:** (11) 1499.
Colinge J, Masselot A, Cusin I, Mahe E, Niknejad A, Argoud-Puy G, Reffas S, Bederr N, Gleizes A, Rey PA, Bougueleret L. 2004. High-performance peptide identification by tandem mass spectrometry allows reliable automatic data processing in proteomics. *Proteomics* **4:** (7) 1977.
Coyne KJ, Burkholder JM, Feldman RA, Hutchins DA, Cary SC. 2004. Modified serial analysis of gene expression method for construction of gene expression profiles of microbial eukaryotic species. *Appl Environ Microbiol* **70:** (9) 5298.
Du Y, Meng FY, Patrie SM, Miller LM, Kelleher NL. 2004. Improved molecular weight-based processing of intact proteins for interrogation by quadrupole-enhanced FT MS/MS. *J Proteome Res* **3:** (4) 801.
Dumur CI, Garrett CT, Archer KJ, Nasim S, Wilkinson DS, Ferreira-Gonzalez A. 2004. Evaluation of a linear amplification method for small samples used on high-density oligonucleotide microarray analysis. *Anal Biochem* **331:** (2) 314.
Fan XL, She YM, Bagshaw RD, Callahan JW, Schachter H, Mahuran DJ. 2004. A method for proteomic identification of membrane-bound proteins containing Asn-linked oligosaccharides. *Anal Biochem* **332:** (1) 178.
Fievet J, Dillmann C, Lagniel G, Davanture M, Negroni L, Labarre J, De Vienne D. 2004. Assessing factors for reliable quantitative proteomics based on two-dimensional gel electrophoresis. *Proteomics* **4:** (7) 1939.
Finehout EJ, Franck Z, Lee KH. 2004. Towards two-dimensional electrophoresis mapping of the cerebrospinal fluid proteome from a single individual. *Electrophoresis* **25:** (15) 2564.
Ghosh D, Krokhin O, Antonovici M, Ens W, Standing KG, Beavis RC, Wilkins JA. 2004. Lectin affinity as an approach to the proteomic analysis of membrane glycoproteins. *J Proteome Res* **3:** (4) 841.
Goodman T, Schulenberg B, Steinberg TH, Patton WF. 2004. Detection of phosphoproteins on electroblot membranes using a small-molecule organic fluorophore. *Electrophoresis* **25:** (15) 2533.
Hajek T, Honys D, Capkova W. 2004. New method of plant mitochondria isolation and sub-fractionation for proteomic analyses. *Plant Sci* **167:** (3) 389.
Halligan BD, Dratz EA, Feng X, Twigger SN, Tonellato PJ, Greene AS. 2004. Peptide identification using peptide amino acid attribute vectors. *J Proteome Res* **3:** (4) 813.
Hart C, Schulenberg B, Patton WF. 2004. Selective proteome-wide detection of hydrophobic integral membrane proteins using a novel fluorescence-based staining technology. *Electrophoresis* **25:** (15) 2486.
Hoffmann GW. 2004. Proteomic analyser with applications to diagnostics and vaccines. *J Theor Biol* **228:** (4) 459.
Hoyt PR, Doktycz MJ. 2004. Optimized beadmilling of tissues for high-throughput RNA production and microarray-based analyses. *Anal Biochem* **332:** (1) 100.
Huang SY, Hsu JL, Morrice NA, Wu CJ, Chen SH. 2004. A convenient method to extract matrix-assisted laser desorption/ionization mass

Copyright © 2005 John Wiley & Sons, Ltd. 

spectrometry spectra from phosphate-containing peptide mixtures. *Proteomics* **4:** (7) 1935.
Islam N, Lonsdale M, Upadhyaya N, Higgins T, Hirano H, Akhurst R. 2004. Protein extraction from mature rice leaves for two-dimensional gel electrophoresis and its application in proteome analysis. *Proteomics* **4:** (7) 1903.
Ito Y, Eiguchi M, Kurata N. 2004. Establishment of an enhancer trap system with *Ds* and GUS for functional genomics in rice. *Mol Genet Genomics* **271:** (6) 639.
Ji W, Zhou WL, Gregg K, Yu N, Davis S, Davis S. 2004. A method for cross-species gene expression analysis with high-density oligonucleotide arrays - Online art. no. e93. *Nucleic Acids Res* **32:** (11) e93.
Ji W, Zhou WL, Gregg KQ, Lindpaintner K, Davis S, Davis S. 2004. A method for gene expression analysis by oligonucleotide arrays from minute biological materials. *Anal Biochem* **331:** (2) 329.
Johnson KL, Muddiman DC. 2004. A method for calculating $^{16}O/^{18}O$ peptide ion ratios for the relative quantification of proteomes. *J Am Soc Mass Spectrom* **15:** (4) 437.
Kaczmarek K, Walczak B, De Jong S, Vandeginste BGM. 2004. Preprocessing of two-dimensional gel electrophoresis images. *Proteomics* **4:** (8) 2377.
Keil A, Shepson PB. 2004. Development of a chemical ionization ion trap mass spectrometric method for trace level determination of molecular halogens. *Anal Chem* **76:** (14) 3951.
Kho Y, Kim SC, Jiang C, Barma D, Kwon SW, Cheng JK, Jaunbergs J, Weinbaum C, Tamanoi F, Falck J *et al*. 2004. A tagging-*via*-substrate technology for detection and proteomics of farnesylated proteins. *Proc Natl Acad Sci U S A* **101:** (34) 12479.
Le Gac S, Carlier J, Camart JC, Cren-Olive C, Rolando C. 2004. Monoliths for microfluidic devices in proteomics. *J Chromatogr B* **808:** (1) 3.
Levander F, Rognvaldsson T, Samuelsson J, James P. 2004. Automated methods for improved protein identification by peptide mass fingerprinting. *Proteomics* **4:** (9) 2594.
Liu CG, Calin GA, Meloon B, Gamliel N, Sevignani C, Ferracin M, Dumitru CD, Shimizu M, Zupo S, Dono M *et al*. 2004. An oligonucleotide microchip for genome-wide microRNA profiling in human and mouse tissues. *Proc Natl Acad Sci U S A* **101:** (26) 9740.
Liu HB, Sadygov RG, Yates JR. 2004. A model for random sampling and estimation of relative protein abundance in shotgun proteomics. *Anal Chem* **76:** (14) 4193.
Liu X, Ma L, Zhang HF, Lu YT. 2004. Determination of single-cell gene expression in *Arabidopsis* by capillary electrophoresis with laser induced fluorescence detection. *J Chromatogr B* **808:** (2) 241.
Ma H, Kafri T. 2004. A single-LTR HIV-1 vector optimized for functional genomics applications. *Mol Ther* **10:** (1) 139.
Macht M, Marquardt A, Deininger SO, Damoc E, Kohlmann M, Przybylski M. 2004. Affinity-proteomics: Direct protein identification from biological material using mass spectrometric epitope mapping. *Anal Bioanal Chem* **378:** (4) 1102.
Mayer M, Yang J, Gitlin I, Gracias DH, Whitesides GM. 2004. Micropatterned agarose gels for stamping arrays of proteins and gradients of proteins. *Proteomics* **4:** (8) 2366.
Mitulovic G, Stingl C, Smoluch M, Swart R, Chervet JP, Steinmacher I, Gerner C, Mechtler K. 2004. Automated on-line two-dimensional nano liquid chromatography tandem mass spectrometry for rapid analysis of complex protein digests. *Proteomics* **4:** (9) 2545.
Moing A, Maucourt M, Renaud C, Gaudillere M, Brouquisse R, Lebouteiller B, Gousset-Dupont A, Vidal J, Granot D, Denoyes-Rothan B *et al*. 2004. Quantitative metabolic profiling by 1-dimensional $^{1}H$-NMR analyses: Application to plant genetics and functional genomics. *Funct Plant Biol* **31:** (9) 889.
Moritz RL, Ji H, Schutz F, Connolly LM, Kapp EA, Speed TP, Simpson RJ. 2004. A proteome strategy for fractionating proteins and peptides using continuous free-flow electrophoresis coupled off-line to reversed-phase high-performance liquid chromatography. *Anal Chem* **76:** (16) 4811.
Murray J, Marusich MF, Capaldi RA, Aggeler R. 2004. Focused proteomics: Monoclonal antibody-based isolation of the oxidative phosphorylation machinery and detection of phosphoproteins using a fluorescent phosphoprotein gel stain. *Electrophoresis* **25:** (15) 2520.
Nakamura T, Dohmae N, Takio K. 2004. Characterization of a digested protein complex with quantitative aspects: An approach based on accurate mass chromatographic analysis with Fourier transform-ion cyclotron resonance mass spectrometry. *Proteomics* **4:** (9) 2558.
Rais I, Karas M, Schagger H. 2004. Two-dimensional electrophoresis for the isolation of integral membrane proteins and mass spectrometric identification. *Proteomics* **4:** (9) 2567.
Reid GE, Roberts KD, Kapp EA, Simpson RJ. 2004. Statistical and mechanistic approaches to understanding the gas-phase fragmentation behavior of methionine sulfoxide containing peptides. *J Proteome Res* **3:** (4) 751.
Resing KA, Meyer-Arendt K, Mendoza AM, Aveline-Wolf LD, Jonscher KR, Pierce KG, Old WM, Cheung HT, Russell S, Wattawa JL *et al*. 2004. Improving reproducibility and sensitivity in identifying human proteins by shotgun proteomics. *Anal Chem* **76:** (13) 3556.
Rose K, Bougueleret L, Baussant T, Bohm G, Botti P, Colinge J, Cusin I, Gaertner H, Gleizes A, Heller M *et al*. 2004. Industrial-scale proteomics: From liters of plasma to chemically synthesized proteins. *Proteomics* **4:** (7) 2125.
Rybak JN, Scheurer SB, Neri D, Elia G. 2004. Purification of biotinylated proteins on streptavidin resin: A protocol for quantitative elution. *Proteomics* **4:** (8) 2296.
Saghatelian A, Jessani N, Joseph A, Humphrey M, Cravatt BF. 2004. Activity-based probes for the proteomic profiling of metalloproteases. *Proc Natl Acad Sci U S A* **101:** (27) 10000.
Saravanan RS, Rose JKC. 2004. A critical evaluation of sample extraction techniques for enhanced proteomic analysis of recalcitrant plant tissues. *Proteomics* **4:** (9) 2522.
Schaefer H, Chervet JP, Bunse C, Joppich C, Meyer HE, Marcus K. 2004. A peptide preconcentration approach for nano-high-performance liquid chromatography to diminish memory effects. *Proteomics* **4:** (9) 2541.
Schulenberg B, Goodman TN, Aggeler R, Capaldi RA, Patton WF. 2004. Characterization of dynamic and steady-state protein phosphorylation using a fluorescent phosphoprotein gel stain and mass spectrometry. *Electrophoresis* **25:** (15) 2526.
Schulenberg B, Patton WF. 2004. Combining microscale solution-phase isoelectric focusing with Multiplexed Proteomics® dye staining to analyze protein post-translational modifications. *Electrophoresis* **25:** (15) 2539.
Shen Y, Tolic N, Masselon C, Pasa-Tolic L, Camp DG, Lipton MS, Anderson GA, Smith RD. 2004. Nanoscale proteomics. *Anal Bioanal Chem* **378:** (4) 1037.
Staes A, Demol H, Van Damme J, Martens L, Vandekerckhove J, Gevaert K. 2004. Global differential non-gel proteomics by quantitative and stable labeling of tryptic peptides with oxygen-18. *J Proteome Res* **3:** (4) 786.
Strittmatter EF, Kangas LJ, Petritis K, Mottaz HM, Anderson GA, Shen YF, Jacobs JM, Camp DG, Smith RD. 2004. Application of peptide LC retention time information in a discriminant function for peptide identification by tandem mass spectrometry. *J Proteome Res* **3:** (4) 760.
Tabuchi M, Baba Y. 2004. Self-contained on-chip cell culture and pretreatment system. *J Proteome Res* **3:** (4) 871.
Thompson DM, King KR, Wieder KJ, Toner M, Yarmush ML, Jayaraman A. 2004. Dynamic gene expression profiling using a microfabricated living cell array. *Anal Chem* **76:** (14) 4098.
Timofeev O, Zhu MM, Gross ML. 2004. Information for proteomics: ESI-MS titration by sodium ions gives the number of carboxylate groups in peptides. *Int J Mass Spectrom* **231:** (2-3) 113.
Vaidyanathan S, Kell DB, Goodacre R. 2004. Selective detection of proteins in mixtures using electrospray ionization mass spectrometry: Influence of instrumental settings and implications for proteomics. *Anal Chem* **76:** (17) 5024.
Wenzl P, Carling J, Kudrna D, Jaccoud D, Huttner E, Kleinhofs A, Kilian A. 2004. Diversity Arrays Technology (DArT) for whole-genome profiling of barley. *Proc Natl Acad Sci U S A* **101:** (26) 9915.
Wu CC, MacCoss MJ, Howell KE, Matthews DE, Yates JR. 2004. Metabolic labeling of mammalian organisms with stable isotopes for quantitative proteomic analysis. *Anal Chem* **76:** (17) 4951.
Wu W, Huang W, Qi RF, Chou YT, Torng E, Watson JT. 2004. ‘Signature sets’, minimal fragment sets for identifying protein disulfide structures with cyanylation-based mass mapping methodology. *J Proteome Res* **3:** (4) 770.
Wu Y, De Kievit P, Vahlkamp L, Pijnenburg D, Smit M, Dankers M, Melchers D, Stax M, Boender PJ, Ingham C *et al*. 2004. Quantitative assessment of a novel flow-through porous microarray for the rapid

Copyright © 2005 John Wiley & Sons, Ltd. 

analysis of gene expression profiles - Online article no. e123. *Nucleic Acids Res* **32:** (15) e123.
Yauk CL, Berndt ML, Williams A, Douglas GR. 2004. Comprehensive comparison of six microarray technologies - Online art. no. e124. *Nucleic Acids Res* **32:** (15) e124.
Zehender H, Le Goff F, Lehmann N, Filipuzzi I, Mayr LM. 2004. SpeedScreen: The "missing link" between genomics and lead discovery. *J Biomol Screen* **9:** (6) 498.
Zhang J, Hu HL, Gao MX, Yang PY, Zhang XM. 2004. Comprehensive two-dimensional chromatography and capillary electrophoresis coupled with tandem time-of-flight mass spectrometry for high-speed proteome analysis. *Electrophoresis* **25:** (14) 2374.
Zhang N, Li N, Li L. 2004. Liquid chromatography MALDI MS/MS for membrane proteome analysis. *J Proteome Res* **3:** (4) 719.
Zilberstein G, Korol L, Bukshpan S, Baskin E. 2004. Parallel isoelectric focusing chip. *Proteomics* **4:** (9) 2533.

### I6 Bioinformatics

Anderssen RS, Wu Y, Dolferus R, Saunders I. 2004. An *a posteriori* strategy for enhancing gene discovery in anonymous cDNA microarray experiments. *Bioinformatics* **20:** (11) 1721.
Andreini C, Bertini I, Rosato A. 2004. A hint to search for metalloproteins in gene banks. *Bioinformatics* **20:** (9) 1373.
Arndt PF, Hwa T. 2004. Regional and time-resolved mutation patterns of the human genome. *Bioinformatics* **20:** (10) 1482.
Azad RK, Borodovsky M. 2004. Probabilistic methods of identifying genes in prokaryotic genomes: Connections to the HMM theory. *Brief Bioinform* **5:** (2) 118.
Bairoch A, Boeckmann B, Ferro S, Gasteiger E. 2004. Swiss-Prot: Juggling between evolution and stability. *Brief Bioinform* **5:** (1) 39.
Beissbarth T, Speed TP. 2004. GOstat: Find statistically overrepresented Gene Ontologies within a group of genes. *Bioinformatics* **20:** (9) 1464.
Belacel N, Cuperlovic-Culf M, Ouellette R. 2004. Fuzzy J-Means and VNS methods for clustering genes from microarray data. *Bioinformatics* **20:** (11) 1690.
Bergemann TL, Laws RJ, Quiaoit F, Zhao LP. 2004. A statistically driven approach for image segmentation and signal extraction in cDNA microarrays. *J Comput Biol* **11:** (4) 695.
Berger DK. 2004. Gene-mining the *Arabidopsis thaliana* genome: Applications for biotechnology in Africa. *S Afr J Bot* **70:** (1) 173.
Berman P, Bertone P, Dasgupta B, Gerstein M, Kao MY, Snyder M. 2004. Fast optimal genome tiling with applications to microarray design and homology search. *J Comput Biol* **11:** (4) 766.
Bluggel M, Bailey S, Korting G, Stephan C, Reidegeld KA, Thiele H, Apweiler R, Hamacher M, Meyer HE. 2004. Towards data management of the HUPO Human Brain Proteome Project pilot phase. *Proteomics* **4:** (8) 2361.
Bourne PE, Westbrook J, Berman HM. 2004. The Protein Data Bank and lessons in data management. *Brief Bioinform* **5:** (1) 23.
Boyle J. 2004. SeqExpress: Desktop analysis and visualization tool for gene expression experiments. *Bioinformatics* **20:** (10) 1649.
Breitling R, Armengaud P, Amtmann A, Herzyk P. 2004. Rank products: A simple, yet powerful, new method to detect differentially regulated genes in replicated microarray experiments. *FEBS Lett* **573:** (1-3) 83.
Chaisson M, Pevzner P, Tang HX. 2004. Fragment assembly with short reads. *Bioinformatics* **20:** (13) 2067.
Chalifa-Caspi V, Yanai I, Ophir R, Rosen N, Shmoish M, Benjamin-Rodrig H, Shklar M, Stein TI, Shmueli O, Safran M *et al.* 2004. GeneAnnot: Comprehensive two-way linking between oligonucleotide array probesets and GeneCards genes. *Bioinformatics* **20:** (9) 1457.
Chen JJ, Delongchamp RR, Tsai CA, Hsueh HM, Sistare F, Thompson KL, Desai VG, Fuscoe JC. 2004. Analysis of variance components in gene expression data. *Bioinformatics* **20:** (9) 1436.
Cheng J, Sun S, Tracy A, Hubbell E, Morris J, Valmeekam V, Kimbrough A, Cline MS, Liu GY, Shigeta R *et al.* 2004. NetAffx gene ontology mining tool: A visual approach for microarray data analysis. *Bioinformatics* **20:** (9) 1462.
Cho H, Lee JK. 2004. Bayesian hierarchical error model for analysis of gene expression data. *Bioinformatics* **20:** (13) 2016.
Churchill GA. 2004. Using ANOVA to analyze microarray data. *Biotechniques* **37:** (2) 173.
Coker JS, Davies E. 2004. Identifying adaptor contamination when mining DNA sequence data. *Biotechniques* **37:** (2) 194.
Costa LD, Barbosa MS, Manoel ETM, Streicher J, Muller GB. 2004. Mathematical characterization of three-dimensional gene expression patterns. *Bioinformatics* **20:** (11) 1653.
Craig R, Beavis RC. 2004. TANDEM: Matching proteins with tandem mass spectra. *Bioinformatics* **20:** (9) 1466.
D'Ascenzo MD, Collmer A, Martin GB. 2004. PeerGAD: A peer-review-based and community-centric web application for viewing and annotating prokaryotic genome sequences. *Nucleic Acids Res* **32:** (10) 3124.
De Hoon MJL, Imoto S, Nolan J, Miyano S. 2004. Open source clustering software. *Bioinformatics* **20:** (9) 1453.
Delongchamp RR, Bowyer JF, Chen JJ, Kodell RL. 2004. Multiple-testing strategy for analyzing cDNA array data on gene expression. *Biometrics* **60:** (3) 774.
Dwight SS, Balakrishnan R, Christie KR, Costanzo MC, Dolinski K, Engel SR, Feierbach B, Fisk DG, Hirschman J, Hong EL *et al.* 2004. *Saccharomyces* genome database: Underlying principles and organisation. *Brief Bioinform* **5:** (1) 9.
Famili AF, Liu GM, Liu ZY. 2004. Evaluation and optimization of clustering in gene expression data analysis. *Bioinformatics* **20:** (10) 1535.
Frith MC, Halees AS, Hansen U, Weng ZP. 2004. Site2genome: Locating short DNA sequences in whole genomes. *Bioinformatics* **20:** (9) 1468.
Ghosh D. 2004. Mixture models for assessing differential expression in complex tissues using microarray data. *Bioinformatics* **20:** (11) 1663.
Golfier G, Dang MT, Dauphinot L, Graison E, Rossier J, Potier MC. 2004. VARAN: a web server for Variability Analysis of DNA microarray experiments. *Bioinformatics* **20:** (10) 1641.
Grasso C, Lee C. 2004. Combining partial order alignment and progressive multiple sequence alignment increases alignment speed and scalability to very large alignment problems. *Bioinformatics* **20:** (10) 1546.
Guda C, Fahy E, Subramaniam S. 2004. MITOPRED: A genome-scale method for prediction of nucleus-encoded mitochondrial proteins. *Bioinformatics* **20:** (11) 1785.
Hanisch D, Sohler F, Zimmer R. 2004. ToPNET - An application for interactive analysis of expression data and biological networks. *Bioinformatics* **20:** (9) 1470.
Haverty PM, Weng ZP. 2004. CisML: An XML-based format for sequence motif detection software. *Bioinformatics* **20:** (11) 1815.
Helman P, Veroff R, Atlas SR, Willman C. 2004. A Bayesian network classification methodology for gene expression data. *J Comput Biol* **11:** (4) 581.
Henrich T, Ramialison M, Segerdell E, Westerfield M, Furutani-Seiki M, Wittbrodt J, Kondoh H. 2004. GSD: A genetic screen database. *Mech Dev* **121:** (7-8) 959.
Hering JA, Innocent PR, Haris PI. 2004. Towards developing a protein infrared spectra databank (PISD) for proteomics research. *Proteomics* **4:** (8) 2310.
Hofacker IL, Stadler PF, Stocsits RR. 2004. Conserved RNA secondary structures in viral genomes: A survey. *Bioinformatics* **20:** (10) 1495.
Hoogland C, Mostaguir K, Sanchez JC, Hochstrasser DF, Appel RD. 2004. SWISS-2DPAGE, ten years later. *Proteomics* **4:** (8) 2352.
Horne AB, Hodgman TC, Spence HD, Dalby AR. 2004. Constructing an enzyme-centric view of metabolism. *Bioinformatics* **20:** (13) 2050.
Huber W, Gentleman R. 2004. matchprobes: A Bioconductor package for the sequence-matching of microarray probe elements. *Bioinformatics* **20:** (10) 1651.
Huson DH, Steel M. 2004. Phylogenetic trees based on gene content. *Bioinformatics* **20:** (13) 2044.
Husson H, Manavalan P, Akmaeva VR, Russo RJ, Cook B, Richards B, Barberio D, Liu DY, Cao XH, Landes GM *et al.* 2004. New insights into ADPKD molecular pathways using combination of SAGE and microarray technologies. *Genomics* **84:** (3) 497.
Ihmels J, Bergmann S, Barkai N. 2004. Defining transcription modules using large-scale gene expression data. *Bioinformatics* **20:** (13) 1993.
Jarman SN. 2004. Amplicon: Software for designing PCR primers on aligned DNA sequences. *Bioinformatics* **20:** (10) 1644.
Ji XL, Yuan Y, Li YD, Sun ZR. 2004. HMMGEP: Clustering gene expression data using hidden Markov models. *Bioinformatics* **20:** (11) 1799.
Jones A, Hunt E, Wastling JM, Pizarro A, Stoeckert CJ. 2004. An object

Copyright © 2005 John Wiley & Sons, Ltd.

model and database for functional genomics. *Bioinformatics* **20:** (10) 1583.
Kahveci T, Ljosa V, Singh AK. 2004. Speeding up whole-genome alignment by indexing frequency vectors. *Bioinformatics* **20:** (13) 2122.
Kauer G, Blocker H. 2004. Analysis of disturbed images. *Bioinformatics* **20:** (9) 1381.
Kerkhoven R, Van Enckevort FHJ, Boekhorst J, Molenaar D, Siezen RJ. 2004. Visualization for genomics: The Microbial Genome Viewer. *Bioinformatics* **20:** (11) 1812.
Kersey PJ, Duarte J, Williams A, Karavidopoulou Y, Birney E, Apweiler R. 2004. The International Protein Index: An integrated database for proteomics experiments. *Proteomics* **4:** (7) 1985.
Kim BS, Rha SY, Cho GB, Chung HC. 2004. Spearman's footrule as a measure of cDNA microarray reproducibility. *Genomics* **84:** (2) 441.
Kimura S, Kawasaki T, Hatakeyama M, Naka T, Konishi F, Konagaya A. 2004. OBIYagns: A grid-based biochemical simulator with a parameter estimator. *Bioinformatics* **20:** (10) 1646.
Konig R, Eils R. 2004. Gene expression analysis on biochemical networks using the Potts spin model. *Bioinformatics* **20:** (10) 1500.
Kreil DP, Karp NA, Lilley KS. 2004. DNA microarray normalization methods can remove bias from differential protein expression analysis of 2D difference gel electrophoresis results. *Bioinformatics* **20:** (13) 2026.
Kumar S, Tamura K, Nei M. 2004. MEGA3: Integrated software for molecular evolutionary genetics analysis and sequence alignment. *Brief Bioinform* **5:** (2) 150.
Li JY, Liu S, Osterman T, Zhang JN, Coppage H, Pedrick N, Witzmann FA. 2004. A software utility for creating interactive maps for 2D gel-based proteomics. *Anal Biochem* **332:** (1) 187.
Li L, Umbach D, Terry P, Taylor JA. 2004. Application of the GA/KNN method to SELDI proteomics data. *Bioinformatics* **20:** (10) 1638.
Matthiesen R, Bunkenborg J, Stensballe A, Jensen ON, Welinder KG, Bauw G. 2004. Database-independent, database-dependent, and extended interpretation of peptide mass spectra in VEMS V2.0. *Proteomics* **4:** (9) 2583.
McHardy AC, Goesmann A, Puhler A, Meyer F. 2004. Development of joint application strategies for two microbial gene finders. *Bioinformatics* **20:** (10) 1622.
Meskauskas A, Lehmann-Horn F, Jurkat-Rott K. 2004. Sight: Automating genomic data-mining without programming skills. *Bioinformatics* **20:** (11) 1718.
Moreira A, Maass A. 2004. TIP: Protein backtranslation aided by genetic algorithms. *Bioinformatics* **20:** (13) 2148.
Nagarajan N, Yona G. 2004. Automatic prediction of protein domains from sequence information using a hybrid learning system. *Bioinformatics* **20:** (9) 1335.
Ng KW, Lawson J, Garner HR. 2004. PathoGene:™ A pathogen coding sequence discovery and analysis resource. *Biotechniques* **37:** (2) 218.
Nilsson RH, Larsson KH, Ursing BM. 2004. galaxie - CGI scripts for sequence identification through automated phylogenetic analysis. *Bioinformatics* **20:** (9) 1447.
Pandey R, Guru RK, Mount DW. 2004. Pathway Miner: Extracting gene association networks from molecular pathways for predicting the biological significance of gene expression microarray data. *Bioinformatics* **20:** (13) 2156.
Parkinson J, Anthony A, Wasmuth J, Schmid R, Hedley A, Blaxter M. 2004. PartiGene - constructing partial genomes. *Bioinformatics* **20:** (9) 1398.
Pavesi G, Mauri G, Iannelli F, Gissi C, Pesole G. 2004. GeneSyn: A tool for detecting conserved gene order across genomes. *Bioinformatics* **20:** (9) 1472.
Perez AJ, Perez-Iratxeta C, Bork P, Thode G, Andrade MA. 2004. Gene annotation from scientific literature using mappings between keyword systems. *Bioinformatics* **20:** (13) 2084.
Poroyko V, Calugaru V, Fredricksen M, Bohnert H. 2004. Virtual-SAGE: A new approach to EST data analysis. *DNA Res* **11:** (2) 145.
Pounds S, Cheng C. 2004. Improving false discovery rate estimation. *Bioinformatics* **20:** (11) 1737.
Prados J, Kalousis A, Sanchez JC, Allard L, Carrette O, Hilario M. 2004. Mining mass spectra for diagnosis and biomarker discovery of cerebral accidents. *Proteomics* **4:** (8) 2320.
Randic M, Lers N, Plavsic D, Basak SC. 2004. On invariants of a 2-D proteome map derived from neighborhood graphs. *J Proteome Res* **3:** (4) 778.
Rangel C, Angus J, Ghahramani Z, Lioumi M, Sotheran E, Gaiba A, Wild DL, Falciani F. 2004. Modeling T-cell activation using gene expression profiling and state-space models. *Bioinformatics* **20:** (9) 1361.
Reich M, Ohm K, Angelo M, Tamayo P, Mesirov JP. 2004. GeneCluster 2.0: An advanced toolset for bioarray analysis. *Bioinformatics* **20:** (11) 1797.
Roberts M, Hunt BR, Yorke JA, Bolanos RA, Delcher AL. 2004. A preprocessor for shotgun assembly of large genomes. *J Comput Biol* **11:** (4) 734.
Robinson AJ, Love CG, Batley J, Barker G, Edwards D. 2004. Simple sequence repeat marker loci discovery using SSR primer. *Bioinformatics* **20:** (9) 1475.
Rogozin IB, Makarova KS, Wolf YI, Koonin EV. 2004. Computational approaches for the analysis of gene neighbourhood in prokaryotic genomes. *Brief Bioinform* **5:** (2) 131.
Saigo H, Vert JP, Ueda N, Akutsu T. 2004. Protein homology detection using string alignment kernels. *Bioinformatics* **20:** (11) 1682.
Schonbach C. 2004. From masking repeats to identifying functional repeats in the mouse transcriptome. *Brief Bioinform* **5:** (2) 107.
Sharma D, Issac B, Raghava GPS, Ramaswamy R. 2004. Spectral Repeat Finder (SRF): Identification of repetitive sequences using Fourier transformation. *Bioinformatics* **20:** (9) 1405.
Sohler F, Hanisch D, Zimmer R. 2004. New methods for joint analysis of biological networks and expression data. *Bioinformatics* **20:** (10) 1517.
Stojanovic N, Dewar K. 2004. Identifying multiple alignment regions satisfying simple formulas and patterns. *Bioinformatics* **20:** (13) 2140.
Stoyanova R, Querec TD, Brown TR, Patriotis C. 2004. Normalization of single-channel DNA array data by principal component analysis. *Bioinformatics* **20:** (11) 1772.
Swertz MA, De Brock EOB, Van Hijum SAFT, De Jong A, Buist G, Baerends RJS, Kok J, Kuipers OP, Jansen RC. 2004. Molecular Genetics Information System (MOLGENIS): Alternatives in developing local experimental genomics databases. *Bioinformatics* **20:** (13) 2075.
Tautz D, Lassig M. 2004. Of statistics and genomes. *Trends Genet* **20:** (8) 344.
Toyoda T, Wada A. 2004. Omic space: Coordinate-based integration and analysis of genomic phenomic interactions. *Bioinformatics* **20:** (11) 1759.
Van Walle I, Lasters I, Wyns L. 2004. Align-m - A new algorithm for multiple alignment of highly divergent sequences. *Bioinformatics* **20:** (9) 1428.
Voit EO, Almeida J. 2004. Decoupling dynamical systems for pathway identification from metabolic profiles. *Bioinformatics* **20:** (11) 1670.
Vracko M, Basak SC. 2004. Similarity study of proteomic maps. *Chemometr Intell Lab Syst* **70:** (1-2) 33.
Wang SJ, Chen JJ. 2004. Sample size for identifying differentially expressed genes in microarray experiments. *J Comput Biol* **11:** (4) 714.
Wei CL, Ng P, Chiu KP, Wong CH, Ang CC, Lipovich L, Liu ET, Ruan YJ. 2004. 5′ Long serial analysis of gene expression (LongSAGE) and 3′ LongSAGE for transcriptome characterization and genome annotation. *Proc Natl Acad Sci U S A* **101:** (32) 11701.
Wendl MC, Yang SP. 2004. Gap statistics for whole genome shotgun DNA sequencing projects. *Bioinformatics* **20:** (10) 1527.
White CN, Chan DW, Zhang Z. 2004. Bioinformatics strategies for proteomic profiling. *Clin Biochem* **37:** (7) 636.
Wu W, Noble WS. 2004. Genomic data visualization on the Web. *Bioinformatics* **20:** (11) 1804.
Xie Y, Jeong KS, Pan W, Khodursky A, Carlin BP. 2004. A case study on choosing normalization methods and test statistics for two-channel microarray data. *Comp Funct Genom* **5:** (5) 432.
Xiong MM, Zhao JY, Xiong H. 2004. Network-based regulatory pathways analysis. *Bioinformatics* **20:** (13) 2056.
Xirasagar S, Gustafson S, Merrick BA, Tomer KB, Stasiewicz S, Chan DD, Yost KJ, Yates JR, Sumner S, Xiao NQ *et al*. 2004. CEBS object model for systems biology data, SysBio-OM. *Bioinformatics* **20:** (13) 2004.
Yuan J, Bush B, Elbrecht A, Liu Y, Zhang T, Zhao WQ, Blevins R. 2004. Enhanced homology searching through genome reading frame predetermination. *Bioinformatics* **20:** (9) 1416.
Zheng J, Close TJ, Jiang T, Lonardi S. 2004. Efficient selection of unique and popular oligos for large EST databases. *Bioinformatics* **20:** (13) 2101.

Copyright © 2005 John Wiley & Sons, Ltd. 
